# Food-Derived Bioactives and the Endothelial Glycocalyx: Chemical Determinants, Molecular Mechanisms, and Translational Evidence

**DOI:** 10.3390/molecules31213832

**Published:** 2026-10-23

**Authors:** Roko Santic, Marko Kumric, Ivan Cvitkovic, Josko Bozic

**Affiliations:** 1 Department of Pathophysiology, University of Split School of Medicine, Soltanska 2A, 21000 Split, Croatia; roko.santic@mefst.hr (R.S.); marko.kumric@mefst.hr (M.K.); 2 Laboratory for Cardiometabolic Research, University of Split School of Medicine, Soltanska 2A, 21000 Split, Croatia; 3 Department of Anesthesiology and Intensive Care, University Hospital of Split, Spinčićeva 1, 21000 Split, Croatia; ivcvitkovic@kbsplit.hr

**Keywords:** endothelial glycocalyx, food bioactives, sulfated polysaccharides, polyphenols, heparan sulfate, bioavailability, endothelial function

## Abstract

Food-derived bioactives can modify the endothelial glycocalyx, but chemical identity, achievable exposure and the meaning of the measured response must be considered together. This narrative review integrates evidence identified through PubMed, Scopus and Web of Science to examine those connections. Experimental studies show reduced loss of surface glycans, altered glycosaminoglycan turnover and changes in layer hydration; selected studies also demonstrate improved permeability or flow-related signaling. These findings support glycocalyx modulation as a biological effect, while distinguishing preservation, swelling and post-injury recovery. Quantitative comparisons reveal substantial gaps between some active culture concentrations and human plasma exposure, compounded by conjugation and uncertain absorption of intact polysaccharides. Surface staining, circulating syndecan-1 and the perfused boundary region measure different processes and cannot individually establish complete restoration. Human trials show favorable surrogate outcomes for some preparations and neutral results for others; multicomponent products and small samples limit ingredient attribution, and responsive patient groups remain unestablished. The contribution of this review is to connect chemical and exposure evidence with what each endothelial endpoint can substantiate. Together, the studies support specific mechanisms of surface-layer modulation but do not yet establish sustained vascular benefit from oral glycocalyx-directed nutrition.

## 1. Introduction

The endothelial glycocalyx links the chemical environment of blood to the physical behavior of the vessel wall. Its proteoglycans, glycosaminoglycans and glycoproteins form a hydrated layer that regulates permeability, limits access to adhesion receptors and helps endothelial cells sense flow [1]. Each of these functions offers a possible route through which nutrition could affect vascular health. Food-derived compounds may change surface-glycan turnover, interact with the layer’s charged polymers, or alter the inflammatory environment in which it is maintained. The challenge is to establish which of these actions occurs at an exposure achievable in humans.

Clinical observations give the glycocalyx potential relevance as an intervention target. Early human studies found lower estimated systemic glycocalyx volume in type 1 diabetes, particularly with microalbuminuria [2], and partial recovery of a reduced volume during lipid-lowering treatment in familial hypercholesterolemia [3]. Experimental acute hyperglycaemia also reduced estimated glycocalyx volume alongside endothelial dysfunction and coagulation activation [4]. In a prospective cohort of 600 people without overt cardiovascular disease, higher baseline sublingual perfused boundary region (PBR) was associated with subsequent cardiovascular events over six years [5]. Whether changing this measurement through nutrition also changes clinical risk remains unknown.

Histological examination of dermal biopsies demonstrated loss of a glycocalyx-associated lectin signal in patients with advanced chronic kidney disease, providing anatomical evidence beyond measurements of circulating fragments [6]. In experimental diabetes, atomic force microscopy detected changes in glycocalyx thickness and endothelial stiffness [7]. High glucose also altered retinal endothelial syndecan-1 and glypican-1 differently, illustrating that injury need not reduce every surface component in parallel [8]. At the murine carotid bifurcation, glycocalyx dimensions varied by arterial region and were smaller in apoE*3-Leiden mice fed an atherogenic diet than in wild-type mice fed normal chow [9]. The affected component and vascular bed therefore matter when selecting a target for intervention.

Diet provides both the metabolic background of these experiments and a potential source of active molecules. Impaired skeletal-muscle glycocalyx barrier properties can occur early during diet-induced obesity [10]. Nevertheless, a blunted adenosine-induced myocardial perfusion response has also been observed without detectable changes in cardiac glycocalyx staining or skeletal-muscle perfused boundary region [11]. The two outcomes can dissociate, making it necessary to measure both when a nutritional intervention is claimed to improve perfusion through the glycocalyx.

**Figure 1:**
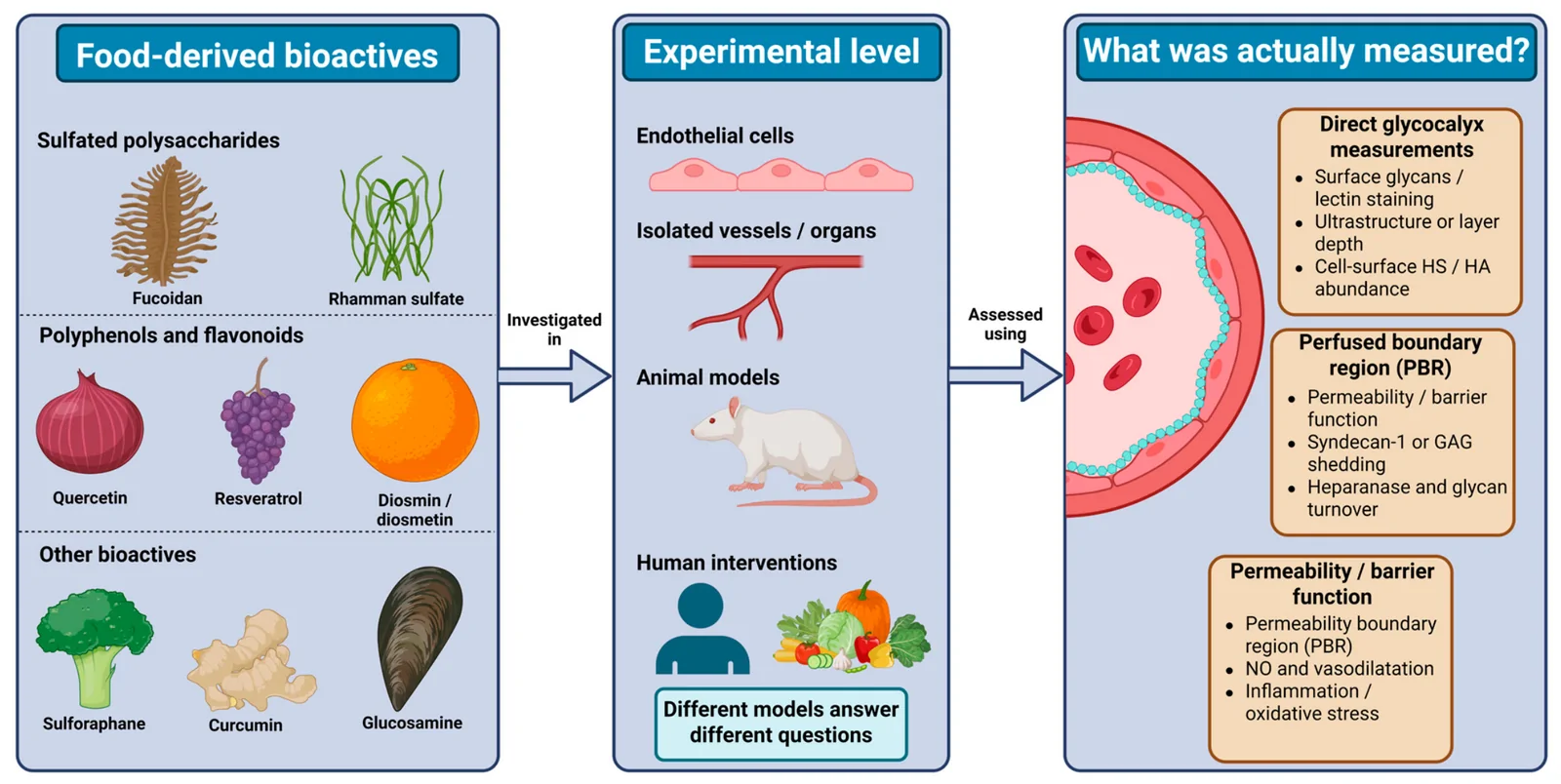
Schematic organization of the evidence linking food-derived bioactives with endothelial glycocalyx-related outcomes. The left panel presents representative compounds grouped as sulfated polysaccharides, polyphenols, flavonoids, and other bioactives. The middle panel shows the experimental levels at which their effects have been investigated, ranging from endothelial-cell cultures and isolated vessels or organs to animal models and human interventions. The right panel distinguishes direct glycocalyx measurements, including surface-glycan staining, ultrastructural assessment and cell-surface heparan sulfate or hyaluronan abundance, from glycocalyx-related measures, such as the perfused boundary region, syndecan-1 or glycosaminoglycan shedding, and glycan turnover. Permeability, nitric oxide-mediated vasodilation, inflammation and oxidative stress are presented as broader endothelial outcomes. These categories are not interchangeable: changes in glycocalyx-related or general endothelial outcomes do not, by themselves, demonstrate structural preservation or restoration of the glycocalyx. The illustrated compounds are representative rather than exhaustive, and the visual sequence does not imply established efficacy or an evidence ranking. GAG, glycosaminoglycan; HA, hyaluronan; HS, heparan sulfate; NO, nitric oxide; PBR, perfused boundary region. Created in BioRender. Kumric, M. (2026) https://BioRender.com/1v1vt0h. Created in https://BioRender.com.

The substances investigated under the heading of natural or nutritional protection differ markedly. A food containing phenolic precursors, a purified citrus flavonoid, a chemically modified marine polysaccharide and an intravenous antioxidant produce different exposures. Earlier reviews addressed nutritional and lifestyle approaches to glycocalyx preservation [12], while a recent review assessed supplements across broader vascular outcomes, including glycocalyx-related measures [13]. The present review builds on that work by linking polymer structure and small-molecule metabolism to experimental concentrations, then examining whether the measured endpoint supports preservation, recovery or only a change in a surrogate. Quantitative exposure comparisons and paired assessment of surface and functional outcomes make this distinction concrete. This focus helps explain which mechanistic findings can inform oral interventions and which still require an exposure bridge. Medicinal extracts and non-oral treatments are included where they clarify that boundary. The central question is whether the chemical species reaching the endothelium can produce the surface change to which vascular benefit is attributed. The representative bioactive classes, experimental levels and distinction between direct glycocalyx measurements, glycocalyx-related measures and general endothelial outcomes are summarized in Figure 1.

## 2. Literature Identification and Scope

The literature for this narrative review was identified through searches of PubMed, Scopus and the Web of Science Core Collection conducted between 28 August and 12 September 2026, with a final update on 19 October 2026. Searches combined glycocalyx and endothelial-surface terms with food, nutrient, extract and bioactive-compound terms. Complementary searches included endothelial heparan sulfate, hyaluronan, syndecans, heparanase and the perfused boundary region. Targeted searches and reference checking supplied supporting literature on chemical characterization, metabolism, bioavailability and measurement validity.

Primary journal articles were eligible for the main synthesis if they examined food-derived bioactives and reported direct or clearly related measures of the endothelial glycocalyx, including surface-glycan measurements, the perfused boundary region, or circulating glycocalyx-associated components. Preprints, conference abstracts, studies of nonvascular glycocalyces, and studies limited to general antioxidant or vascular effects without a relevant glycocalyx endpoint were excluded from the main synthesis. Selection was based on titles and available abstracts, followed by assessment of accessible full texts. Mechanistic, pharmacokinetic and measurement-validation studies were retained as supporting context where relevant. Where access was limited to an abstract, interpretation was restricted to explicitly reported information. Findings were synthesized qualitatively, with attention to neutral results, potentially overlapping cohorts and the distinction between nutritional exposure and pharmacological administration.

## 3. Endothelial Glycocalyx Biology and Its Assessment

The endothelial glycocalyx forms a hydrated, carbohydrate-rich interface between blood and the vessel wall. Its properties arise from membrane-associated molecules and incorporated plasma proteins. Transmembrane syndecans and glycosylphosphatidylinositol-anchored glypicans carry glycosaminoglycan chains, particularly heparan sulfate (HS); hyaluronan (HA) associates with receptors including CD44, while glycoproteins contribute terminal sialic acids. Within this dynamic layer, HS-associated binding sites retain proteins involved in anticoagulant and antioxidant defense, and the hydrated mesh regulates access of circulating cells and macromolecules to the endothelial membrane [1]. The contribution of each constituent depends on the function examined. Preserving one component does not necessarily restore every glycocalyx function. Selective glycan digestion produced different changes in thickness and small-solute diffusion, including reduced diffusion after HS removal. A thinner layer can thus retain substantial resistance to solute movement [14].

Mechanotransduction links this molecular organization to vascular function. Enzymatic HS removal suppressed shear-induced nitric oxide (NO) production while preserving the bradykinin response, locating the defect in flow sensing [15]. Depletion of HS, HA or sialic acids impaired shear-induced NO, whereas prostacyclin responses remained intact [16]. Glypican-1 knockdown inhibited endothelial NO synthase (eNOS) activation, while syndecan-1 depletion preferentially disrupted cytoskeletal remodeling under flow [17]. Syndecan-1 knockout also altered Akt, paxillin and RhoA responses and increased inflammatory adhesion [18]. HS-dependent flow-induced NO production was subsequently demonstrated in intact rat microvessels [19].

The glycocalyx also influences NO availability through redox mechanisms. In isolated porcine arteries, HS degradation was accompanied by extracellular superoxide dismutase loss and increased oxidative NO consumption [20]. Sialic-acid removal attenuated flow-induced nuclear factor erythroid 2-related factor 2 (NRF2) signaling in human endothelial cells, placing the glycocalyx upstream of antioxidant responses [21]. Read alongside hydrogen peroxide-induced HS shedding in glomerular endothelial cultures [22], these results suggest reciprocal effects between oxidative injury and glycocalyx disruption, with the two directions tested in separate experimental models.

### 3.1. Injury and Reconstitution

Glycocalyx injury involves glycan cleavage, proteoglycan shedding and oxidative fragmentation. In experimental sepsis, heparanase activation degraded pulmonary endothelial HS and facilitated neutrophil adhesion and lung injury; genetic and pharmacological inhibition supported a causal contribution [23]. Cerebral ischemia experiments implicated acrolein-mediated proheparanase activation and increased hyaluronidase-1 expression, linking reactive carbonyl stress with glycan-degrading enzymes [24]. Oxidative effects also depend on the reactive species. In cell-free experiments, hydroxyl and carbonate radicals fragmented HA and chondroitin sulfate, whereas nitrogen dioxide did not produce equivalent fragmentation [25]. Hydrogen peroxide induced HS shedding and increased albumin passage in human glomerular endothelial cultures, connecting oxidative exposure with a barrier defect [22]. Localized hydrogen peroxide can also contribute to physiological dilation in coronary resistance vessels [26]. Its role depends on the vascular bed and signaling context, so lowering a bulk oxidant signal is insufficient evidence that endothelial function has recovered.

Protease-dependent loss affects both matrix retention and signaling. Matrix metalloproteinase inhibition reduced glycan shedding and leukocyte adhesion in inflamed rat venules [27]. Neuraminidase exposure has been linked to ADAM17-dependent glypican-1 shedding and impaired flow-mediated dilation; separate work identified ADAM17-mediated CD44 cleavage as another route to impaired shear sensing [28,29]. These surface substrates connect proteolysis directly to loss of mechanosensor function. For bioactive compounds, reduced inflammatory signaling and preserved surface mechanosensing should be reported as distinct outcomes.

Reconstitution requires synthesis, assembly and a supportive extracellular environment. Sphingosine-1-phosphate limited syndecan-1 shedding through receptor-dependent suppression of metalloproteinase activity; subsequent experiments showed recovery of surface glycans through a phosphoinositide 3-kinase (PI3K)-dependent process [30,31]. Pulmonary repair after experimental HS depletion involved fibroblast growth factor receptor signaling and the HS-synthesis enzyme EXT1. Circulating HS fragments could support this response, which was delayed in sepsis [32]. Tie2 activation was reported to promote reconstitution after septic or enzymatic endothelial injury [33]. Whether orally consumed compounds engage these repair processes remains an exposure-dependent question.

Shear itself stimulates HA incorporation alongside HA release, allowing shedding to coexist with renewal [34]. Flow-regulated glycocalyx formation also depends on Krüppel-like factor 2 (KLF2)-associated HA synthase trafficking and glucose-derived biosynthetic substrates [35]. Similar endpoint measurements may consequently reflect protection, replacement or altered turnover. Distinguishing them requires attention to when an intervention begins relative to injury and when the surface is measured.

### 3.2. What Glycocalyx Measurements Can Establish

Experimental conditions shape both the surface layer and its response. One direct comparison found a hydrodynamically substantial layer in vivo but little corresponding layer in standard endothelial cultures [36]. Perfusate composition matters as well: albumin and hydroxyethyl starch supported different NO-mediated flow responses in isolated hearts despite their colloidal properties [37].

Imaging and force measurements capture different features (Table 1). Nonaqueous fixation preserved a substantial macromolecular coat on endothelial surfaces [38]. Fluorescence and electron microscopy gave markedly different absolute thickness estimates in the same microvessels, although neuraminidase-induced structural changes tracked increased water and albumin permeability [39]. Atomic force microscopy (AFM) separates surface coverage, effective layer length and underlying endothelial stiffness, which changed at different stages of experimental diabetes [7]. The resulting dimensions and elastic properties depend on the force-curve model and cannot be interchanged directly across analytical approaches [40].

Red-cell exclusion measurements enabled early estimates of human microvascular glycocalyx dimensions [41]. Current sublingual PBR measurements quantify the lateral region accessible to erythrocytes, calculated as half the difference between the perfused diameter and median red-cell column width. Dynamic PBR additionally adjusts the estimate for its relationship with red-cell velocity in feeding vessels. Both are model-derived indices of red-cell access, not anatomical thickness. Vessel-size selection, image quality and pressure artifacts also affect interpretation [42]. One reproducibility study found poor repeatability [43]; another found improved reliability through averaging repeated recordings and documented effects of meals, coffee and smoking [44]. Standardized acquisition is therefore particularly relevant to nutrition studies. Regional differences add a separate limitation. Glycocalyx composition varies across vascular territories, and experimental endotoxemia affected pulmonary and cremasteric layers differently [23,45]. A sublingual response cannot establish equivalent changes in coronary, cerebral or glomerular glycocalyx.

Circulating markers integrate release from tissues with systemic clearance. Renal transplantation studies identified possible tubular contributions to circulating syndecan-1 [46]. Renal clearance of syndecan-1 and HS varied in burn patients, affecting interpretation of their plasma concentrations [47]. HA also originates from extracellular matrices, and blocking hepatic HA uptake established the contribution of clearance through the hyaluronan receptor for endocytosis [48]. Differences between assays further limit comparisons of absolute syndecan-1 concentrations [49]. The analyte, assay and sampling interval need to be reported before discordant results can be attributed to different biological responses. Lower soluble concentrations may accompany protection, but rebuilding is better supported by agreement between a surface measurement and a relevant functional outcome, interpreted with the limitations of each method.

**Table 1:** Interpretation of commonly used glycocalyx measurements.

Measurement	What Is Measured	Principal Limitation	References
Electron or fluorescence microscopy	Labeled surface architecture and apparent depth	Fixation, probe specificity and image analysis influence dimensions	[38,39]
AFM	Force-derived layer length, coverage and mechanics	Model assumptions and underlying cell stiffness affect estimates	[7,40]
Sublingual PBR	Lateral erythrocyte penetration into the perfused boundary	Flow-dependent surrogate; not anatomical thickness or an organ-wide measurement	[42]
Circulating syndecan-1, HS or HA	Soluble antigen or glycan concentration	Tissue source, renal/hepatic clearance and assay differences confound release	[46,47,48]
Microvascular permeability	Water conductance, albumin reflection or solute passage	Functional outcome integrates glycocalyx and other vessel-wall determinants	[39]

AFM, atomic force microscopy; HA, hyaluronan; HS, heparan sulfate; PBR, perfused boundary region. Changes measured by different methods should not be treated as equivalent quantities.

Evidence should be judged by the claim it can support. A change in soluble fragments or PBR first establishes modulation of that particular readout. Component-specific surface measurements or imaging provide more direct evidence of composition or architecture, but require controls for hydration, probe specificity and sampling. A restoration claim is stronger when an established defect reverses alongside a relevant barrier or flow-dependent function in the same model. Durable improvement in patient-relevant outcomes requires separate clinical evidence. These are complementary evidential requirements rather than interchangeable measurements or a validated numerical score; concordant structural and functional changes do not by themselves prove mediation. Table 2 applies this distinction to paired PBR and syndecan-1 results.

## 4. Chemical Identity and Endothelial Exposure

A food source does not define the chemical species reaching the endothelial glycocalyx. Whole foods, standardized extracts and purified compounds can differ in composition, release during digestion and subsequent metabolism. Bioaccessibility describes the fraction released from the food matrix and remaining available for intestinal absorption after digestion, and should be distinguished from systemic bioavailability [50]. Simulated digestion can characterize this release but does not establish the circulating concentration presented to the endothelium. In human quercetin studies, glucosides were absorbed more rapidly than rutinosides, and comparisons of onion, isolated glycosides and buckwheat preparations showed that glycoside identity could outweigh the matrix effect [51,52]. Olive-oil studies demonstrated absorption and urinary recovery of phenolic derivatives, including after moderate, sustained oil consumption [53,54]. Urinary recovery supports dietary exposure without establishing the concentration of unconjugated hydroxytyrosol at the endothelial surface. Extract dose, constituent dose and circulating active species require separate descriptions (Table 3).

Matrix effects should be separated from glycoside effects. In a 12-participant crossover study, buckwheat tea and isolated rutin, each providing 200 mg quercetin equivalents, produced different peak timing (4.3 versus 7.0 h) and measured peak plasma concentrations (0.6 versus 0.3 µg/mL). Conversely, an onion supplement and isolated quercetin-4′-O-glucoside at 100 mg quercetin equivalents showed no significant pharmacokinetic difference [52]. Only glucuronidated quercetin was detected. Thus, a matrix may modify the circulating exposure profile, but does not invariably increase bioavailability; these findings neither quantify unbound endothelial exposure nor establish glycocalyx target engagement.

For many polyphenols, the major circulating species differ from the molecules commonly applied to cultured endothelial cells. After onion ingestion, individual quercetin glucuronides and sulfates reached submicromolar concentrations; enzymatic hydrolysis before analysis would conceal this diversity [55]. Ingestion of 500 mL green tea produced several circulating conjugates alongside detectable unchanged gallated catechins, including epigallocatechin gallate at a peak concentration of approximately 55 nM in one feeding study [56]. Exposure comparisons must account for both conjugation and persistence of parent compounds. The tested species, concentration, exposure duration and analytical definition of “total” compound determine how closely an experiment represents oral exposure.

Tracer studies distinguish recovery of compound-derived material from exposure to the intact molecule. After 60 mg radiolabeled epicatechin, approximately 82% of the administered label was recovered in urine, but systemic exposure involved conjugated and microbial metabolites rather than detectable unchanged epicatechin [57]. A study using 500 mg labeled cyanidin-3-glucoside estimated relative bioavailability of 12.4% from urinary and breath recovery [58]. Further analysis of the same participants identified numerous degradation products and conjugates with distinct concentration–time profiles [59]. These complementary analyses represent one tracer cohort, not independent replications, and recovered label does not represent intact anthocyanin availability. Microbial conversion adds interindividual variability: ellagic-acid interventions produced different urolithin metabolite profiles across participants [60]. Microbial conversion and host conjugation together determine the chemical forms available after food consumption. For endothelial experiments, this favors testing measured circulating metabolites at concentrations attained in humans, rather than relying solely on the parent compound named in a food or supplement [61].

Resveratrol illustrates the distinction between absorption and parent-compound bioavailability. With a 25 mg oral dose, absorption was substantial but unchanged plasma resveratrol remained very low because of extensive metabolism [62]. A phase-I study using doses up to 5 g produced a mean parent-compound peak of approximately 2.4 µM at the highest dose, with substantially greater conjugate exposure [63]. Sustained exposure to tens of micromolar parent resveratrol in cell experiments therefore requires justification beyond these oral pharmacokinetic data. Plasma measurements do not establish endothelial uptake, local deconjugation or glycocalyx binding. “Unconjugated” and “unbound” describe different properties: chemical identity and protein binding.

For diosmin, both the formulation and analytical method affect exposure comparisons. An early human study detected diosmetin after oral diosmin, rather than the administered glycoside [64]. Micronization increased radiolabel recovery, which measures absorption of compound-derived material, not necessarily intact diosmin [65]. A later formulation comparison measured plasma diosmetin after β-glucuronidase treatment, so reported concentrations included diosmetin released from conjugates [66]. Comparisons with direct endothelial exposure to diosmin or diosmetin must account for these analytical differences; a shared ingredient name establishes neither formulation equivalence nor equivalence of circulating species.

Curcumin formulation studies show why exposure gains require chemical and functional context. After 10–12 g doses, conjugates were readily detected, whereas unconjugated curcumin was detected in only one participant [67]. A crossover comparison linked improved availability to post-digestive solubility, while detecting conjugated rather than free curcumin [68]. Another quantified parent curcuminoids alongside reduced and conjugated metabolites: total exposure differed across formulations, although unconjugated curcumin exposure did not differ significantly at the administered product doses [69]. Dose normalization and metabolite selection affect the interpretation of these comparisons. A micellar preparation increased measured curcuminoid exposure without reducing IL-6 or TNF-α responses in an ex vivo lipopolysaccharide (LPS) model [70]. Inferring glycocalyx protection from improved pharmacokinetics still requires functional evidence.

Processing affects precursor conversion as well as absorption. Sulforaphane exposure was higher after raw than cooked broccoli in a human feeding study [71]. In a broccoli-sprout crossover trial, a beverage containing myrosinase-generated sulforaphane produced greater and less variable urinary metabolite recovery than a glucoraphanin-rich beverage relying on conversion after ingestion [72]. Broccoli-extract mass or glucoraphanin content alone omits a key determinant of delivered exposure: the availability of active myrosinase or preformed sulforaphane.

Polysaccharides require characterization beyond a compound name or source species. A characterized *Laminaria hyperborea* fucoidan and chemically modified derivatives differed in molecular mass, sulfation and biological properties [73]. Distinct *Saccharina japonica* fractions and their hydrolysates also show why source species alone cannot establish equivalence [74]. Comparisons require molecular-mass distributions, monosaccharide composition, sulfate content and substitution pattern, linkage information where available, and purity, usually established through complementary chromatographic and spectroscopic methods [75]. These specifications identify the tested material without establishing a glycocalyx mechanism; efficacy claims for a starting material cannot simply be transferred to a depolymerized or desulfated preparation.

Assay specificity also limits interpretation of oral polysaccharide exposure. Human fucoidan studies detected antibody-reactive material in plasma or urine after ingestion [76,77]. The earlier plasma study conditioned its absorption interpretation on the assumption that the measured material was intact galactofucan; the larger urinary study reported cross-reactivity with another fucoidan source. Immunoreactivity supports exposure to recognized epitopes without establishing the size, integrity or endothelial concentration of the administered polymer. Intravenous animal experiments bypass this uncertainty and cannot alone justify equivalent effects from oral supplementation.

Chemical antioxidant capacity is not a surrogate for glycocalyx preservation. Electron-transfer and radical-trapping assays describe reactions under their particular conditions [78]. Polyphenols can generate hydrogen peroxide in culture media, producing responses attributable partly to extracellular oxidation rather than the proposed cellular target [79]. Sulforaphane acts through a different mechanism: electrophilic interaction with Kelch-like ECH-associated protein 1 (KEAP1) thiols provides a basis for inducing NRF2-regulated cytoprotective responses without requiring direct radical scavenging [80]. Curcumin requires controls for instability and optical or other assay interference [81]. Purity also matters: bacterial lipoproteins and lipopolysaccharides accounted for much of the macrophage activation in several botanical preparations [82]. This finding supports measuring endotoxin and relevant co-extractives before assigning activity to a purified polysaccharide or botanical constituent, without establishing contamination of any particular glycocalyx study.

An exposure–effect assessment should begin with the intended oral preparation and the chemical species responsible for the experimental response. Three questions then follow: is that species detected in humans, does its concentration–time profile overlap the active experimental exposure, and is the relevant endothelial response demonstrated? Dietary exposure, supplement-derived exposure and pharmacological administration should be distinguished by the administered preparation and measured exposure rather than by compound class. Peak plasma concentration (Cmax) can identify an obvious concentration mismatch; area under the concentration–time curve (AUC) and persistence are additionally relevant when effects require prolonged treatment. Similar AUC values would not, however, establish equivalence between transient postprandial exposure and continuously replenished culture medium. Comparisons should distinguish unchanged parent compounds, individual metabolites and measurements obtained after enzymatic deconjugation [55,56,57,58,59,63,66,69]. Where available, protein binding in plasma and experimental medium should also be considered: nominal culture concentration and total plasma concentration are not measurements of the same unbound exposure. Concentration–response data for the same chemical species would permit a more informative exposure margin, but such a ratio remains descriptive without evidence of endothelial uptake or target engagement. The metabolite, diosmetin, glucosamine and fucoidan examples below illustrate exposure-informed selection, large nominal gaps and analytically unresolved comparisons. Missing pharmacokinetic information should remain an explicit uncertainty, not be replaced by assumptions.

**Table 3:** Chemical and exposure distinctions relevant to interpreting glycocalyx experiments.

Material	Chemical Determinant	Exposure Implication	References
Quercetin preparations	Glucoside versus rutinoside; conjugation	Absorption kinetics differ; circulating conjugates require consideration.	[52,55]
Anthocyanins	Parent pigments and degradation products	Tracer recovery includes metabolites; it is not intact-pigment bioavailability.	[58,59]
Curcumin formulations	Solubility, reduction and conjugation	Higher total exposure need not mean higher unconjugated exposure.	[69]
Diosmin preparations	Particle size; conversion to diosmetin	Radiolabel and deconjugated measurements do not quantify intact diosmin.	[65,66]
Broccoli preparations	Glucoraphanin, myrosinase and preformed sulforaphane	Processing changes precursor conversion and delivered exposure.	[72]
Fucoidans	Size distribution, sulfation, composition and purity	Antibody recognition alone does not establish intact systemic polymer.	[75,76]

## 5. Sulfated Polysaccharides

### 5.1. Structural Determinants and HS-Dependent Interactions

Marine polysaccharides differ in backbone structure, branching and sulfation; “fucoidan” does not identify a single reproducible molecule. A fraction from *Saccharina japonica* (reported as *Laminaria japonica*) contained predominantly α-(1→3)-linked fucose, with α-(1→4) linkages, fucose/galactose branches and position-dependent sulfation [83]. Rhamnan sulfate from the edible green alga *Monostroma nitidum* instead contains a branched rhamnose-rich backbone, including differently sulfated 1→2- and 1→3-linked residues [84]. These polymers can reproduce selected interactions of heparan sulfate (HS) without reproducing its sequence or biosynthesis. Molecular-mass distribution, monosaccharide composition, sulfate position, branching and purification define each preparation. At 10 µg/mL, *Ascophyllum nodosum* fucoidan fractions with number-average masses of 4.9 and 26.6 kDa differed in endothelial migration and network formation. Their sugar composition and sulfate/fucose ratios also differed, preventing attribution to molecular size alone [85].

Early experiments focused on endothelial HS synthesis. A 21 kDa xylofucoglucuronan from *Spatoglossum schroederi* stimulated endothelial synthesis of antithrombotic HS [86]. A distinct 24 kDa xylogalactofucan also stimulated HS synthesis without appreciable anticoagulant activity in the reported assays [87]. The related 21.5 kDa galactofucan contained a partially sulfated galactose backbone with fucose/xylose branches; desulfation abolished both stimulation of endothelial HS synthesis and delayed antithrombotic activity after intravenous administration [88]. Conditioned-medium experiments implicated secreted HS, leaving reconstruction of the luminal layer unproven. These sulfate-dependent responses provide mechanistic comparisons; the algal source requires independent confirmation of food use. Route further constrained activity: fucan A lacked antithrombotic activity after oral administration despite activity through other routes [89].

Deliberate chemical modification produces preparations distinct from extracted or fractionated native polymers. Depolymerization changes chain length and potentially the distribution of binding sites; oversulfation can change activity qualitatively, precluding attribution of a derivative’s effects to the native polysaccharide in a seaweed meal. Oversulfated fucoidan suppressed lipopolysaccharide-induced plasminogen activator inhibitor-1 release from human endothelial cells, whereas native fucoidan had negligible activity; active fragments spanned several molecular-weight ranges [90]. Heparitinase and anti-HS-proteoglycan experiments implicated an HS-dependent endotoxin response; de novo glycocalyx assembly was not demonstrated. Under fibroblast growth factor 2 (FGF-2) stimulation, oversulfated fucoidan instead increased PAI-1 release while inhibiting endothelial migration and tube formation [91]. Conversely, low-molecular-weight preparations enhanced HS-dependent FGF-2 responses [92] and promoted vascular endothelial growth factor 165 (VEGF165) interactions with VEGFR-2 and neuropilin-1 [93]. Preparation and ligand thus help determine the response. In another study, low-molecular-weight fucoidan promoted angiogenesis while increasing heparanase expression/activity and altering syndecan expression and shedding [94]. Angiogenic remodeling and glycocalyx preservation are distinct endpoints; this study also rules out describing all fucoidans as heparanase inhibitors.

These comparisons support a conditional structure–activity relationship rather than a rule that more sulfate or lower molecular mass is necessarily more protective. Sulfate groups provide anionic interaction sites, while chain length, branching and linkage geometry can influence how those sites are presented to proteins. The strongest mechanistic interpretation is therefore altered engagement of HS-binding ligands or regulation of endogenous HS synthesis, rather than presumed direct binding of one anionic polysaccharide to another. Loss of HS-synthesis stimulation after desulfation demonstrates a sulfate requirement for that particular galactofucan response [88]; it does not identify an optimal sulfation degree or prove reconstruction of the luminal layer. Likewise, the contrasting FGF-2 responses to oversulfated and low-molecular-weight preparations, together with enhanced VEGF165 receptor interactions, show that ligand context and polymer preparation can change the direction of activity [91,92,93]. Molecular mass cannot be isolated as the causal determinant when fractions also differ in sugar composition and sulfate content [85]. Although linkage configuration and branching define the polymer scaffold [83,84], their independent contribution to endothelial glycocalyx outcomes has not been established by these comparisons. Future structure–activity experiments should therefore compare chemically characterized derivatives while holding other properties as constant as possible, and pair protein-interaction measurements with surface-component and functional endpoints. Such designs would distinguish structural determinants of ligand modulation from determinants of glycocalyx preservation.

### 5.2. Glycocalyx Preservation and Post-Injury Recovery

Injury models have examined endothelial surface glycans alongside barrier function. In primary pulmonary microvascular endothelial cells, co-exposure to serum from patients with severe COVID-19 and *L. japonica* fucoidan (10 µg/mL for 24 h) attenuated the loss of lectin-detected surface glycans and thrombomodulin. Electrical barrier function and cell-surface coagulation measurements also improved [95]. Patients did not receive fucoidan. A complementary study found recovery of mechanically assessed glycocalyx properties when a fucoidan-containing mixture was applied after dialysis-serum or heparinase injury. This response was attenuated by extracellular signal-regulated kinase (ERK)-pathway inhibition and inhibition of vesicular transport [96]. Although isolated fucoidan reproduced an acute effect, the orally tested mixture also contained glucosamine, hyaluronan and antioxidant ingredients, preventing attribution of its in vivo effect to fucoidan alone.

Oral low-molecular-weight fucoidan at 40 mg/kg/day for seven weeks preserved aortic and glomerular glycocalyx measurements in diabetic db/db mice without significantly changing blood glucose. Supporting culture experiments used 25–200 µg/mL fucoidan during 24 h exposure to 56 mM glucose and implicated neuraminidase-2 through expression, binding and perturbation experiments [97]. Both overexpression and knockdown damaged the glycocalyx, suggesting that regulated neuraminidase activity, rather than maximal inhibition, was required. Oral activity in these db/db mice does not identify the route linking ingestion to the endothelial response. NEU2 binding and cellular perturbations support a direct mechanism under the experimental exposure, but do not show that intact orally ingested polymer reaches vascular NEU2 at an active concentration. Indirect intestinal or microbiota-dependent effects remain possible. Fucoidan directly inhibited recombinant heparanase in a separate glomerulonephritis study, yet intravenous treatment produced no clear reduction in albuminuria despite changes in inflammatory outcomes [98]. In isolated perfused rat hearts, administration during reperfusion preserved ultrastructural glycocalyx features and reduced microvascular obstruction [99]. This exposure bypassed intestinal absorption and cannot establish oral human efficacy.

### 5.3. Other Sulfated Polymers

Rhamnan sulfate inhibits endothelial tissue-factor expression and von Willebrand factor release, supporting anti-inflammatory and antithrombotic actions without directly establishing glycocalyx protection [100]. Oral pretreatment reduced vascular leakage after endotoxin challenge in mice and preserved lung lectin staining and syndecan-4 abundance [101]. The preparation averaged approximately 500 kDa. Whole-lung syndecan-4 measurements did not establish an endothelial source. In a separate atherosclerosis study, rhamnan sulfate enhanced endothelial barrier function in the presence of glycocalyx-degrading enzymes and reduced LDL deposition. Dietary administration reduced atherosclerosis in ApoE-deficient mice, with several stronger responses in females. Growth-factor binding and suppressed inflammatory signaling offer additional explanations for the antiatherosclerotic effect, limiting its attribution to glycocalyx preservation [102]. Another oral study preserved vascular Robo4 expression and improved lipid and inflammatory outcomes, providing complementary vascular evidence rather than an independent demonstration of glycocalyx reconstruction [103].

Sodium spirulan isolated from *Spirulina platensis* increased release of perlecan and biglycan from bovine endothelial cell layers, mostly with intact glycosaminoglycan chains and core proteins, although partial core-protein degradation was also detected [104]. Mobilization of these proteoglycans was proposed to increase anticoagulant activity in the surrounding fluid. Because perlecan and biglycan also occupy extracellular matrix compartments, these experiments neither localize the released material to the luminal glycocalyx nor establish improved barrier integrity.

### 5.4. Oral Exposure and Indirect Intestinal Pathways

Absorption studies further delimit the relevance of cell-culture potency to oral exposure. Intestinal-cell and rat studies detected uptake of *Cladosiphon okamuranus* fucoidan, including accumulation in intestinal and hepatic macrophages, but transport was limited and experimental conditions influenced uptake [105]. A mouse pharmacokinetic comparison reported different exposure for native and low-molecular-weight *S. japonica* preparations using serum fucose measurements; these do not selectively quantify intact polymers [106]. After volunteers consumed 100 g of mozuku containing approximately 1 g of fucoidan, serum fucoidan remained undetectable in the ten participants who provided blood samples, despite urinary detection in four [107]. The 10–200 µg/mL concentrations applied directly in the injury models above therefore lack a demonstrated human exposure counterpart. Differences in source polymer, food matrix and assay prevent calculation of a valid exposure ratio. Intestinal barrier or microbial effects offer an indirect route to lower systemic inflammatory exposure [108], but their contribution to the reported glycocalyx responses remains unresolved.

A microbiota-mediated route is compatible with the broader conceptual treatment of food-medicine homologous polysaccharides as multifunctional materials [109]. It remains an alternative hypothesis for the oral glycocalyx response, rather than a demonstrated explanation. Experimental precedent comes from a separate T2DM rat study: fucoidan improved intestinal-barrier and metabolic outcomes, and fecal microbiota transfer from treated donors reproduced improvements in recipients [110]. This supports a microbiota-dependent contribution in that model, but neither establishes direct polymer binding at the vascular surface nor proves the mechanism in the db/db glycocalyx study. Polymer-specific exposure measurements combined with microbiota perturbation or transfer and vascular endpoints would help distinguish these routes.

Across these experiments, sulfated polysaccharides alter HS synthesis and turnover, modify growth-factor interactions and attenuate loss of surface-associated glycans during injury. Sequential treatment also demonstrates recovery of mechanical surface properties in a defined model. These findings establish local endothelial responses to particular preparations. Oral animal studies extend their vascular relevance, while leaving the active circulating species and the contribution of indirect intestinal mechanisms uncertain.

## 6. Small Molecules and Nutrient Interventions

The experimental literature concerns chemically different exposures: intact food constituents, phenolic metabolites, standardized extracts, and purified compounds administered at pharmacological doses. The stilbene resveratrol and the flavonoids epicatechin and quercetin, which belong to different subclasses, have all been tested against the same endothelial nanomechanical endpoints [111]. Shared responses do not make these compounds or their metabolites interchangeable. Their reported effects also encompass distinct processes: glycocalyx hydration, reduced shedding and recovery of surface components.

### 6.1. Phenolic Metabolites and Physical Layer Responses

To examine metabolites encountered after food consumption, Cutler et al. treated diabetic human aortic endothelial cells with sulfate-conjugated benzoic and vanillic acid derivatives at 75–700 nM, hydroxyhippuric acid at 3 µM and hippuric acid at 5 µM. These concentrations were selected to reflect human plasma after blueberry consumption. After three days, incorporation of radiolabeled sulfate into cell-surface glycosaminoglycans increased and monocyte adhesion declined. Released glycosaminoglycan abundance and the HS/chondroitin-sulfate ratio remained unchanged; low radiolabel recovery prevented detailed disaccharide analysis of the cell-associated fraction [112]. This design addresses circulating chemical identity, although daily metabolite addition during three days of culture differs from a transient postprandial profile. Blueberry feeding also changed glycosaminoglycan composition in whole rat aortas [113], including those of spontaneously hypertensive rats. Whole-aorta extraction cannot localize this response to the luminal glycocalyx [114].

Acute nanomechanical responses are consistent with altered surface-layer hydration. At 10 µg/mL, hawthorn extract WS 1442 increased apparent layer thickness and reduced stiffness in excised mouse aortas within 30 min. Separate cultured-cell measurements found no increase in surface HS abundance. In endothelial monolayers exposed to the extract for four days, its reduction of sodium permeability disappeared after heparinase treatment [115]. These experiments distinguish rapid physical reorganization from the longer exposure used to test barrier behavior. Resveratrol, epicatechin and quercetin also induced glycocalyx swelling in cultured endothelial cells; inhibition of the cystic fibrosis transmembrane conductance regulator (CFTR) prevented swelling while leaving cortical softening intact [111]. Minute-scale swelling and three-day sulfate incorporation into cell-associated GAGs [112] therefore describe different processes. A larger layer need not represent new assembly, and exposure to medicinal hawthorn extract differs from consumption of its fruit.

### 6.2. Flavonoids and Other Purified Natural Products

Diosmin, the glycosylated flavone related to diosmetin, was tested after partial carotid ligation in male ApoE-deficient mice. Treatment at 50 mg/kg/day orally or intraperitoneally for seven days began 72 h after surgery. Both routes increased wheat germ agglutinin (WGA)-detected luminal coverage and apparent layer thickness and reduced CD68-positive cell accumulation. Plasma hyaluronan decreased significantly only with intraperitoneal treatment; changes in p-eNOS/total eNOS and vessel dimensions were not significant. This small experiment (3–4 mice per group) supports structural and anti-inflammatory effects after injury without establishing restored vasomotor function or whether glycocalyx recovery mediates reduced inflammation [116].

Diosmetin was tested during progression of sterile inflammation in mice with myeloid Mcpip1 deletion. Daily oral gavage at 40 mg/kg from three to six months of age increased liver WGA staining and lowered circulating syndecan-1, with less hepatic inflammatory infiltration despite persistent systemic inflammation. WGA fluorescence in fixed liver sections, interpreted as sinusoidal endothelial glycocalyx, does not resolve luminal thickness or barrier function. Supporting TNF-α co-exposure experiments used 20–40 µM diosmetin for 24 h to examine syndecan-1 mRNA in primary mouse liver sinusoidal endothelial cells and protein abundance in human umbilical vein endothelial cells [117]. By comparison, a single 500 mg formulated diosmin dose in healthy men produced a mean plasma peak of 50.3 ng/mL, approximately 0.17 µM diosmetin equivalents, measured after β-glucuronidase treatment [66]. The culture concentrations exceeded even this deconjugated signal by more than two orders of magnitude. Because the human measurement includes conjugate-derived diosmetin, it does not establish equivalent unchanged exposure. The mouse findings support protection during disease progression at the tested dose; benefit at ordinary dietary exposure remains unresolved.

In late endothelial progenitor cells exposed to advanced glycation products, resveratrol preserved cell-associated syndecan-4 ectodomain immunoreactivity, interpreted by the authors as reduced shedding, and improved tube formation alongside Akt/eNOS phosphorylation. The published figures label resveratrol doses in mmol/L; without clarification of those units, a reliable comparison with oral exposure is not possible [118]. Apigenin at 10 µg/mL (approximately 37 µM) attenuated soluble syndecan-1 release from human retinal microvascular endothelial cells under diabetes-mimetic conditions [119]. These studies measure retained cellular protein and a released ectodomain, respectively; neither directly establishes restoration of mature surface architecture.

Curcumin studies use different delivery routes and endpoints. A transdermal formulation reduced albumin and dextran leakage in endotoxemic rats, but the reported permeability experiments did not directly measure glycocalyx reconstruction [120]. Oral Curcuma longa supplementation in adults with type 2 diabetes lowered circulating syndecan-1, syndecan-4, and hyaluronan [121]. Permeability and circulating markers provide complementary evidence of endothelial effects across these formulations, while the repair mechanism remains unresolved. The clinical trial is discussed below.

In acute inflammatory injury models, oral pretreatment with the xanthone glucoside mangiferin reduced lung and kidney albumin permeability in endotoxemic mice, accompanied by preservation of syndecan-1 and occludin and lower matrix metalloproteinase-9 (MMP-9) expression [122]. Crocin preserved HS and syndecan-4 in LPS-exposed experimental systems while reducing cathepsin L, heparanase, and MMP-9 expression [123]. Astilbin pretreatment likewise reduced heparanase activity and circulating HS in an LPS-induced lung-injury model [124]. Reduced activity or expression of extracellular-degrading enzymes accompanies broader suppression of inflammation and oxidative stress, making their individual contributions difficult to separate. Pretreatment designs also leave reversal of established injury unresolved.

Among purified medicinal-plant constituents, the lotus-embryo alkaloid neferine was tested both before and after LPS exposure. Post-injury treatment increased endothelial HS and syndecan-1 staining relative to spontaneous recovery, alongside lower mitochondrial oxidant signals. The mouse recovery experiment used oral neferine for three days beginning six hours after LPS, whereas prevention was tested with seven days of pretreatment [125]. Berberine reduced HS/syndecan-1 shedding in experimental endotoxemia and promoted recovery of these components after injury [126]. Under low shear stress, it additionally preserved hyaluronan through AMP-activated protein kinase (AMPK)-associated regulation of p47phox, hyaluronidase-2 and hyaluronan synthase 2 [127]. Ginsenoside Rg1 preserved WGA staining and proteoglycan expression in high-glucose-exposed endothelial cells, increased electrical resistance, and reduced albumin passage [128]. These defined natural products were tested pharmacologically; equivalent vascular exposure from their source plants or foods has not been demonstrated.

### 6.3. Vitamins, Precursors and Other Nutrients

Nutrient effects vary with cell type, dose and timing. In cholesterol-fed guinea pigs, vitamin E was associated with preservation of the endothelial surface glycocalyx and lower abnormal vascular permeability [129]. At 0.1 mM, ascorbate reduced cell-associated proteoglycans in cultured retinal endothelial cells while increasing them in pericytes [130]. In septic mice, subcutaneous ascorbate given immediately after cecal ligation and puncture reduced syndecan-1 elevation and tetrahydrobiopterin (BH4) oxidation; administration delayed for 12 h did not improve survival. The 200 mg/kg dose and circulating syndecan-1 endpoint limit extrapolation to oral nutrition or direct surface reconstruction [131]. Small human studies of high-dose intravenous ascorbate have reported early changes in sublingual microcirculation/PBR [132] and attenuation of syndecan-1 increases in a post hoc acute respiratory distress syndrome (ARDS) analysis [133]. Such parenteral exposures provide no direct evidence about dietary vitamin C.

Glucosamine provides a precursor rationale for GAG-containing supplements, but the concentration and tissue compartment matter. Bovine aortic endothelial cells exposed to 2.5 mM glucosamine for 16 h produced more perlecan in the matrix and medium and transported less LDL across the monolayer [134]. This nominal concentration is about 250 times the approximately 10 µM plasma peak reported after 1500 mg/day oral crystalline glucosamine sulfate for three days [135]. Plasma concentration does not directly measure intracellular hexosamine-pathway flux, but the gap limits extrapolation to luminal repair at supplement-derived exposure or to N-acetylglucosamine mixtures. Mineral intake can alter GAG structure without increasing its abundance: manganese-supplemented rats had less aortic HS and HA and less-sulfated HS than adequately supplied controls [136]. Whole-aorta extraction left the cellular source unresolved.

Amino-acid interventions have also been tested. In a preeclampsia-like mouse model, gestational L-citrulline supplementation improved estimated systemic glycocalyx volume and vascular function, with effects also reported postpartum; this remains preclinical evidence rather than a treatment recommendation for pregnancy [137]. In diabetic mice, a diet containing doubled aspartate and asparagine reduced albuminuria and increased glomerular WGA staining, with qualitatively stronger microvascular eNOS immunostaining. The co-intervention precludes attribution solely to aspartate, while glomerular lectin staining does not resolve endothelial surface thickness independently of other glycoconjugates [138].

Functional responses can also diverge within an experiment. Butyrate increased endothelial, heparinase-sensitive antithrombin binding, yet also increased ICAM-1, combining potentially anticoagulant and activation-related effects [139]. Linoleic acid exposure reduced proteoglycan synthesis and the anionic properties of cell-associated HS proteoglycans while increasing albumin transfer in porcine endothelial cultures [140]. Neither benefit nor harm from habitual food intake can be inferred from these culture exposures. Interpretation depends on identifying the chemical exposure, locating the surface change and relating it to endothelial function.

## 7. Human Intervention Evidence

Several small trials report improvements in sublingual glycocalyx measurements, whereas others using related preparations are neutral (Table 4). The central clinical question is whether this variation identifies responsive patient groups or reflects differences in trial design and measurement. Current studies cannot resolve that question. Their contribution is best judged from prespecified comparisons between randomized groups, with vascular function assessed alongside the glycocalyx outcome.

### 7.1. Multicomponent Glycocalyx Supplements

Several trials evaluated preparations containing fucoidan, glucosamine, hyaluronan and additional antioxidant ingredients. Any treatment effect belongs to the complete formulation. In a double-blind pilot trial, 23 older adults were randomized to Endocalyx Pro at six capsules daily (3712 mg/day) or placebo for 12 weeks; all completed treatment. Overall PBR was unchanged relative to placebo in the 22 participants with measurements, as was conventional brachial flow-mediated dilation (FMD) in all 23 participants. The FMD mean ratio was 1.16 (95% confidence interval 0.77–1.74), compatible with considerable uncertainty rather than proof of equivalence. FMD normalized to shear-rate area under the curve favored supplementation in 20 participants (mean ratio 2.41; *p* = 0.001), although baseline shear differed between groups and the normalized response declined in the placebo group. This analysis was described in the methods. Separately, a post hoc analysis excluding antihypertensive users found a capillary-PBR signal in 16 participants; overall PBR remained neutral, as did conventional FMD in the corresponding 17-person subset. These findings do not establish a responsive clinical subgroup or show that glycocalyx restoration mediated the normalized FMD response [141]. A subsequent sex analysis used 22 participants from the same trial; it raises a hypothesis about differential responses rather than providing independent replication. Neither sex showed a significant supplement-versus-placebo PBR difference in that analysis [142].

The diabetes results are particularly instructive. Among predominantly male veterans with type 2 diabetes, 24 participants were randomized and 22 analyzed after eight weeks of Endocalyx supplementation at six capsules daily. Each capsule contained 106.25 mg fucoidan, 375 mg glucosamine sulfate, 17.5 mg hyaluronan and a 120 mg blend of superoxide dismutase and polyphenols. Despite high adherence, there was no improvement over placebo in PBR, brachial or femoral FMD, arterial stiffness, or insulin-stimulated leg microvascular perfusion. The concurrent benefit in diabetic mice makes the absence of a human response particularly informative [143].

The paired experiments also differed in exposure and readout. Female db/db mice received 100 mg/kg/day for four weeks, whereas veterans received 3712.5 mg/day for eight weeks. These nominal doses do not establish comparable systemic exposure to any constituent. Mouse responses were assessed by AFM in aortic explants and FMD in isolated mesenteric arteries, whereas human assessment combined sublingual PBR with conduit-artery and skeletal-muscle functional outcomes [143]. Species, sex distribution, disease background and vascular-bed differences therefore remain candidate modifiers, rather than established explanations. The human course was longer in calendar time, so shorter treatment alone cannot explain its neutral result. Although the pilot sample leaves uncertainty about modest effects, concordant neutrality across several human vascular outcomes argues against dismissing the findings as a measurement-specific failure.

A separate three-arm trial randomized 56 South Asian Surinamese adults with type 2 diabetes to supplementation, a fasting-mimicking diet, or placebo. Four capsules daily supplied the same stated constituent quantities per capsule as the veterans’ preparation. At three months, dynamic PBR favored supplementation (group-by-time interaction, *p* < 0.001), but adjusted baseline values were 2.74 µm in the supplement arm and 2.19 µm with placebo. The adjusted within-arm changes were −0.73 and +0.28 µm, respectively; −0.73 µm was not the treatment-versus-placebo effect estimate. Static PBR changed little with supplementation (−0.04 µm), compared with worsening on placebo (+0.16 µm). The initial imbalance and different responses of these metrics temper interpretation. Albuminuria and glycocalyx-degrading enzyme activities did not improve. The dietary arm improved metabolic measures without reproducing the supplement’s dynamic-PBR response. Favorable dynamic PBR and microvascular health scores were still reported three months after supplementation stopped [144]. The contrast with the neutral veterans’ trial requires replication; it does not establish which clinical characteristics, dose or treatment duration explain the difference.

Trials in inflammatory conditions have reported more favorable PBR findings. In psoriasis, 56 patients receiving biological treatment were randomized and 50 completed four months. PBR decreased by 9.95% with supplementation versus 0.87% with placebo, with a significant time-by-treatment interaction (*p* = 0.009). Skin severity improved similarly in both groups, making background biological treatment essential context [145]. In a separate study beginning 14 days after COVID-19 onset in patients managed as outpatients, 62 participants were randomized and 57 analyzed. Four-month PBR changes were −6.8% versus −1.3% (between-group *p* = 0.032), accompanied by favorable vascular measurements. The timing places this study in early recovery rather than established long COVID [146]. Neither inflammatory-disease trial was designed to establish a reduction in clinical vascular events.

Urinary peptidomic findings in 44 South Asian Surinamese participants extend the earlier diabetes trial. A reduction in the HF2 classifier within the supplement group does not demonstrate fewer heart-failure events or a statistically established treatment effect between groups; CAD160 and CKD273 classifiers were unchanged [147].

### 7.2. Defined Polyphenol Preparations and Other Nutritional Interventions

A randomized crossover trial in 30 patients with chronic coronary disease compared hydroxytyrosol-enriched olive-oil capsules, supplying 10 mg hydroxytyrosol daily, with olive-oil-containing placebo capsules for one month each. The reported PBR percentage change favored enrichment (−1.9% versus +5.2%, *p* = 0.025), alongside improved FMD. The comparator supports an inference about enrichment rather than olive oil consumption generally. Nevertheless, the short treatment periods and predominantly male sample limit conclusions about durability and generalizability; the absence of an explicitly described washout leaves possible carryover unresolved [148].

A six-month study in metabolic dysfunction-associated steatotic liver disease analyzed 60 participants receiving placebo or a preparation delivering 240 mg coenzyme Q10 daily. The active product also contained choline, milk-thistle and artichoke ingredients, selenium and several vitamins. PBR decreased within the active group, but the reported between-group comparisons did not establish a significant PBR advantage. Its contribution is limited to an exploratory observation for a multicomponent product [149].

A 2026 trial of *Curcuma longa* extract in type 2 diabetes reported lower post-treatment syndecan-1, syndecan-4 and hyaluronan concentrations with 1200 mg/day of an extract standardized to 97% curcuminoids for three months. Between-group *p* values were 0.001, 0.040 and 0.002, respectively. However, 107 participants were randomized and only 76 analyzed: 46 in the extract group and 30 receiving placebo. Eighteen placebo recipients withdrew consent, and gastrointestinal events caused further withdrawals in both groups. This differential attrition substantially weakens causal interpretation despite the article’s intention-to-treat terminology. Circulating-marker changes also do not establish endothelial structural restoration, and plasma NRF2 or sirtuin 1 (SIRT1) concentrations cannot demonstrate activation of these pathways within endothelial cells [121].

In the FLALOC trial, 46 patients with persistent post-COVID fatigue completed 90 days of an epicatechin-enriched cacao-husk preparation or placebo. Two daily capsules supplied 80 mg of free epicatechin. Fatigue and functional outcomes favored treatment, while circulating syndecan-1 decreased within the active group. The syndecan-1 figure reports paired within-group tests, leaving the treatment-versus-placebo difference in change unresolved. Whether the symptom improvement was mediated by a glycocalyx effect remains an open question [150].

Trials of other food-related interventions have been neutral. In 18 healthy young men, two weeks of pomegranate extract with a total phenolic content of 720 mg/day expressed as gallic-acid equivalents did not improve PBR or syndecan-1 responses to repeated sprint cycling relative to an untreated control. The small sample, absence of a placebo and limited evidence of glycocalyx injury during the exercise challenge constrain interpretation; the result cannot be generalized to patients with established microvascular disease [151]. In ProFOS, 41 adults with type 1 diabetes and albuminuria entered a randomized crossover study and 35 completed it. Twelve weeks of a synbiotic preparation did not improve the PBR-derived glycocalyx estimate or the primary albuminuria endpoint compared with placebo. Gut-directed nutritional mechanisms therefore remain insufficient grounds for assuming glycocalyx benefit [152].

### 7.3. Clinical Interpretation

Taken together, the trials show that some oral formulations can lower sublingual PBR over weeks to months. The South Asian diabetes, psoriasis and early post-COVID trials support modifiability of this microvascular readout, while neutral findings in older adults and veterans show that related preparations do not consistently produce it. Circulating-marker studies identify changes in soluble glycocalyx-associated material without establishing that the endothelial surface has recovered. Where vascular function also improves, parallel changes do not demonstrate that the glycocalyx mediated the benefit. The common clinical finding is therefore a capacity to alter selected surrogate outcomes, with no established class effect or consistent link to tissue perfusion and durable clinical benefit. The contributions of formulation, population and methods to conflicting results remain unknown. Replication with defined preparations and prospectively specified outcomes is the immediate priority.

Between-trial heterogeneity should be considered along four axes: baseline impairment and room for improvement; formulation and exposure duration; population background, including concomitant vascular treatment; and outcome definition and analysis. The baseline dynamic-PBR imbalance in the South Asian trial, its separation from static PBR, and biological treatment in psoriasis illustrate why apparently similar supplement trials are not interchangeable [144,145]. Measurement timing, repeated acquisition and whether benefit reflects improvement on treatment or deterioration on placebo further affect interpretation [42,43,44,144]. These factors could modify measured responses, but none has been established as the cause of the contrasting results. Harmonized protocols and prespecified tests of interaction with baseline impairment are needed before proposing responsive subgroups.

Declared interests also merit consideration. The South Asian diabetes trial reported a relationship with the measurement-technology manufacturer [144]. The psoriasis trial disclosed technology-related interests while explicitly stating no commercial interest in the tested supplement [145]. Manufacturer provision of study products also occurred in the neutral veterans’ trial [143]. Replication by independent groups would help assess both the intervention and the measurement procedure. Table 4 summarizes the populations, interventions, treatment durations, and principal glycocalyx-related findings of selected randomized human trials.

**Table 4:** Selected randomized human interventions with glycocalyx-related outcomes.

Population and Preparation	Sample; Exposure	Main Findings vs. Comparator	Interpretation and Reference
Older adults; combined supplement	23 randomized; 6 capsules/day, 12 weeks	Overall PBR (n = 22) and conventional FMD neutral	Normalized FMD improved; capillary-PBR signal post hoc [141]
Type 2 diabetes, veterans; combined supplement	24 randomized; 6 capsules/day, 8 weeks	PBR and vascular function unchanged	22 analyzed [143]
South Asian Surinamese diabetes; combined supplement	56 across three arms; 4 capsules/day, 3 months	Dynamic PBR favored supplement	Enzyme activities and albuminuria unchanged [144]
Psoriasis; combined supplement	56 randomized; 4 months	PBR −9.95% versus −0.87%	50 completers; concurrent biologics [145]
Early post-COVID; combined supplement	62 randomized; 4 months	PBR −6.8% versus −1.3%	57 analyzed; early recovery [146]
Coronary disease; enriched olive oil	30; crossover, 1 month each	PBR change favored hydroxytyrosol enrichment	Olive-oil comparator; short exposure [148]
Type 2 diabetes; curcuminoid extract	107 randomized; 3 months	Lower syndecans and hyaluronan	Only 76 analyzed; differential attrition [121]
Type 1 diabetes; synbiotic	41 randomized; crossover, 12 weeks each	No glycocalyx benefit versus placebo	35 completed; albuminuria also unchanged [152]

PBR, perfused boundary region; lower values indicate less erythrocyte penetration. Selected trials illustrate different preparations and outcome types; additional interventions appear in the text. Counts distinguish randomized and analyzed populations. Mixtures do not establish ingredient efficacy; circulating markers and PBR represent different measurements.

## 8. Interpretation and Priorities for Translation

### 8.1. Vascular Bed and Background Diet

Glycocalyx measurements should be interpreted in the vascular bed and metabolic setting in which they were obtained. In experimental obesity, loss of endothelial Kir2.1 flow sensitivity was linked to altered glycocalyx biophysics, and restoring channel expression rescued flow-induced vasodilation [153]. Subsequent comparisons showed different glycocalyx and channel responses in visceral and subcutaneous adipose arteries [154]. This regional variation helps explain why sublingual, organ-specific and circulating measurements may give different answers to the same intervention.

The direction of a structural change also requires functional corroboration. An atherogenic diet reduced arterial glycocalyx dimensions alongside lower expression of biosynthetic components and impaired vasomotor responses in rats [155]. In another experiment, high salt reduced microvascular density without changing mean PBR within diet groups, whereas a Western diet lowered PBR [156]. A subsequent study detected Western-diet-associated increases in estimated thickness from the first week, with a contribution from hyaluronan, despite impaired acetylcholine-mediated vasodilation [157]. Six-hour fasting normalized the thickness estimate while leaving PBR unchanged [158]. Greater measured thickness is therefore an ambiguous treatment target unless barrier- or flow-dependent function improves as well. Background diet and measurement timing can change the apparent direction of benefit.

Human biomarker studies show a similar dissociation. After bariatric surgery, circulating syndecan-1 decreased while hyaluronan increased, despite improvement in several other circulating endothelial markers [159]. The anatomical sources were unresolved. Marker selection should therefore follow the component and process the intervention is expected to affect.

### 8.2. Establishing a Clinically Meaningful Effect

Exposure and mechanism need to be tested in the same experimental context. For small molecules, this means testing the circulating species actually produced by the intended food or formulation. For polysaccharides, it means reporting molecular-mass distribution and composition rather than relying on a class name or a single nominal dose. An effect after injection is pharmacologically informative; its relevance to an oral preparation depends on demonstrating the required exposure or an indirect mechanism. Comparisons between active and inactive fractions, parent compounds and metabolites, and native and modified polymers can be more revealing than another experiment using a broadly labeled extract.

Experimental rescue should also be separated from prevention. Pretreatment before an acute inflammatory insult can demonstrate that an intervention changes susceptibility to injury. A clinical claim of restoration requires treatment after an established defect and evidence that the abnormality reverses. Measurements of surface components should be accompanied, where feasible, by permeability or flow-dependent function in the same preparation. Enzyme inhibition, receptor perturbation or genetic manipulation can test necessity, but a change in protein abundance alone does not establish that the protein mediates the response.

Clinical safety requires direct assessment at the intended exposure. In LOVIT, high-dose intravenous vitamin C increased the composite of death or persistent organ dysfunction in adults with sepsis; the study does not establish harm from ordinary dietary vitamin C [160]. Ten adjudicated cases of turmeric-associated liver injury were reported by the Drug-Induced Liver Injury Network. That series identifies a supplement-associated safety concern without estimating incidence or the risk of culinary turmeric [161].

Clinical trials should use reproducible products and prespecified comparisons that match the proposed mechanism. Baseline glycocalyx status, background diet, concomitant drugs and renal function can influence both the opportunity for improvement and the behavior of circulating markers. A trial of a mixture can establish an effect of that mixture; isolating the contribution of an ingredient requires an appropriate comparator. Between-group estimates with confidence intervals are more informative than separate statements that one group improved and another did not. Differential withdrawal, adherence, repeated outcomes and the handling of missing observations deserve particular attention in the small trials available so far.

Ingredient attribution requires designs matched to the question. A randomized dose-ranging study of a chemically defined single agent, with measured circulating species and prespecified safety and vascular outcomes, can establish whether an active exposure is attainable. For mixtures, a factorial trial restricted to selected components can estimate constituent effects and test selected interactions when adequately powered. With many ingredients, a dismantling design comparing the complete formulation with an otherwise matched preparation lacking one candidate component may be more feasible. This estimates that component’s contribution within the mixture, rather than its efficacy alone. Dose escalation characterizes exposure and safety but does not itself establish ingredient causality or synergy. Formulation, capsule burden and vehicle should be matched to avoid changing several determinants simultaneously.

Baseline PBR predicted cardiovascular events in an observational cohort [5], but the clinical meaning of lowering PBR with treatment is still unknown. Improved dynamic PBR did not coincide with lower albuminuria in the South Asian Surinamese trial [144], whereas the veterans’ trial was neutral for both PBR and vascular function [143]. The next clinical advance would be to show that a sustained surface-layer response accompanies a relevant functional benefit. Establishing whether baseline impairment identifies patients more likely to respond would also make future trials more informative. Neither prognostic association nor a favorable exploratory subgroup provides that answer.

## 9. Conclusions

The experimental evidence converges on three effects: preservation of surface glycans during injury, altered glycosaminoglycan turnover, and changes in glycocalyx hydration and organization. Selected studies connect these changes to lower permeability, reduced cell adhesion or improved flow-related signaling. Post-injury recovery has also been demonstrated in specific models, but cannot be inferred from pretreatment or simultaneous exposure alone.

The translational constraint is the connection between the active material, endothelial exposure and the outcome measured. Polymer fractions with different compositions are not interchangeable, and small-molecule effects at high culture concentrations cannot be assigned to dietary exposure without accounting for circulating metabolites. Human studies show that oral formulations can change some glycocalyx-related surrogates, but they have not established a consistent functional benefit, efficacy of individual mixture components, or reduced clinical events. The field therefore has credible evidence of surface-layer modulation and a narrower, still unresolved question: which orally achievable chemical exposures produce a sustained improvement in the vascular function that the glycocalyx supports?

**Figure 2:**
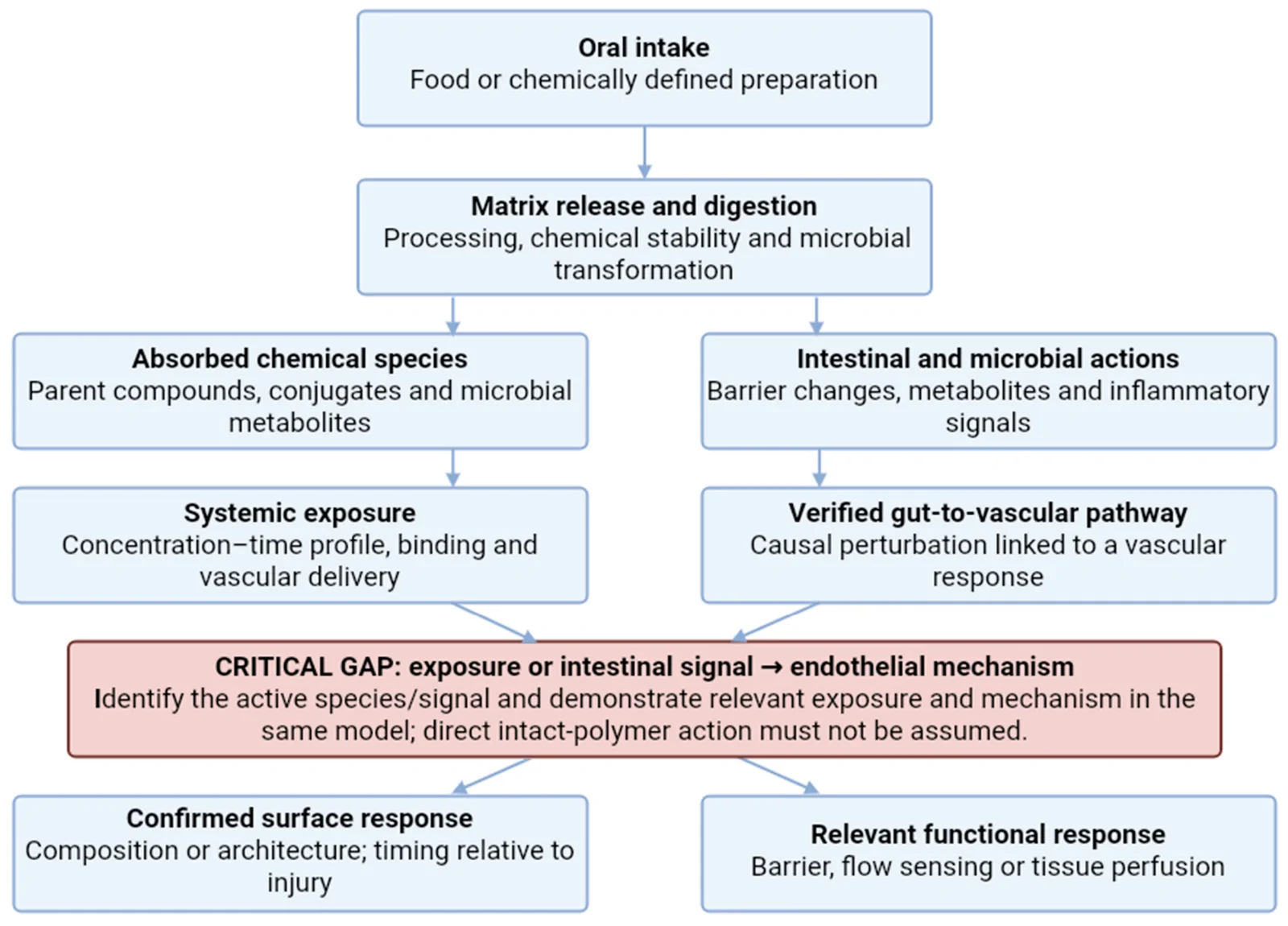
From oral intake to an interpretable endothelial glycocalyx effect. The two branches distinguish exposure to absorbed chemical species from indirect intestinal or microbiota-dependent actions. Dashed arrows denote links requiring explicit verification for the preparation and model, rather than established efficacy. The central gap is demonstration of relevant exposure or an intestinal signal coupled to the proposed endothelial mechanism. Surface and functional responses provide complementary evidence; their association does not alone establish mediation or clinical benefit. The diagram is an assessment framework, not a completed causal pathway for every bioactive. Created in BioRender. Kumric, M. (2026) https://BioRender.com/hog4ykr. Created in https://BioRender.com.

Future research should focus on two complementary priorities. First, oral dose–exposure–response studies should identify the circulating species produced by a chemically defined preparation and test whether achievable concentrations engage the proposed endothelial mechanism. Second, standardized, repeated glycocalyx measurements should be paired prospectively with a relevant barrier-, perfusion-, or flow-dependent functional endpoint, using prespecified between-group analyses. These steps should precede larger efficacy trials, so that a favorable surrogate change is linked to an interpretable exposure and sustained vascular benefit. Figure 2 summarizes the oral-to-endothelial chain and its principal evidential gap: identifying the active chemical exposure or verified intestinal signal that engages the proposed mechanism and produces a sustained surface and functional response.

## Figures and Tables

**Table 2:** Interpretation of paired PBR and circulating syndecan-1 responses.

Paired Finding	Possible Interpretation	Required Checks and Limit of Inference
Lower PBR; lower syndecan-1	Concordant changes in regional erythrocyte access and circulating material.	Check timing, flow, clearance and functional outcomes. Agreement supports modulation, not complete structural restoration.
Lower PBR; unchanged or higher syndecan-1	A regional access or hydration response may coexist with shedding or turnover elsewhere.	Repeat standardized PBR recordings; examine sampling kinetics and tissue sources. Neither result invalidates the other.
Unchanged or higher PBR; lower syndecan-1	Reduced release or altered clearance may occur without improved sublingual erythrocyte exclusion.	Assess renal/hepatic function, assay consistency and vascular-bed relevance. Lower marker concentration alone does not demonstrate repair.
Discordant results on repeat assessment	Different processes, vascular territories, kinetics or measurement effects remain possible.	Reassess acquisition and assay quality, prespecified analysis and independent surface/function outcomes. Do not average unlike endpoints into a repair estimate.

PBR, perfused boundary region. “Lower” refers to the prespecified intervention-versus-comparator change. Interpretations are hypotheses requiring corroboration; PBR and syndecan-1 are not measurements of the same biological quantity.

## Data Availability

No new data were created or analyzed in this study. Data sharing is not applicable to this article.

## Abbreviations

The following abbreviations are used in this manuscript:

ADAM17	a disintegrin and metalloproteinase 17
AFM	atomic force microscopy
eNOS	endothelial nitric oxide synthase
FGF-2	fibroblast growth factor 2
FMD	flow-mediated dilation
GAG	glycosaminoglycan
HA	hyaluronan
HS	heparan sulfate
LPS	lipopolysaccharide
MMP-9	matrix metalloproteinase 9
NRF2	nuclear factor erythroid 2-related factor 2
PBR	perfused boundary region
WGA	wheat germ agglutinin

## References

[1] Reitsma S., Slaaf D.W., Vink H., Van Zandvoort M.A.M.J., Oude Egbrink M.G.A. (2007). The Endothelial Glycocalyx: Composition, Functions, and Visualization. Pflug. Arch.-Eur. J. Physiol..

[2] Nieuwdorp M., Mooij H.L., Kroon J., Atasever B., Spaan J.A.E., Ince C., Holleman F., Diamant M., Heine R.J., Hoekstra J.B.L. (2006). Endothelial Glycocalyx Damage Coincides With Microalbuminuria in Type 1 Diabetes. Diabetes.

[3] Meuwese M.C., Mooij H.L., Nieuwdorp M., Van Lith B., Marck R., Vink H., Kastelein J.J.P., Stroes E.S.G. (2009). Partial Recovery of the Endothelial Glycocalyx upon Rosuvastatin Therapy in Patients with Heterozygous Familial Hypercholesterolemia. J. Lipid Res..

[4] Nieuwdorp M., Van Haeften T.W., Gouverneur M.C.L.G., Mooij H.L., Van Lieshout M.H.P., Levi M., Meijers J.C.M., Holleman F., Hoekstra J.B.L., Vink H. (2006). Loss of Endothelial Glycocalyx During Acute Hyperglycemia Coincides With Endothelial Dysfunction and Coagulation Activation In Vivo. Diabetes.

[5] Ikonomidis I., Thymis J., Simitsis P., Koliou G.-A., Katsanos S., Triantafyllou C., Kousathana F., Pavlidis G., Kountouri A., Polyzogopoulou E. (2022). Impaired Endothelial Glycocalyx Predicts Adverse Outcome in Subjects Without Overt Cardiovascular Disease: A 6-Year Follow-up Study. J. Cardiovasc. Trans. Res..

[6] Koch J., Hijmans R.S., Ossa Builes M., Dam W.A., Pol R.A., Bakker S.J.L., Pas H.H., Franssen C.F.M., Van Den Born J. (2021). Direct Evidence of Endothelial Dysfunction and Glycocalyx Loss in Dermal Biopsies of Patients With Chronic Kidney Disease and Their Association With Markers of Volume Overload. Front. Cell Dev. Biol..

[7] Targosz-Korecka M., Jaglarz M., Malek-Zietek K.E., Gregorius A., Zakrzewska A., Sitek B., Rajfur Z., Chlopicki S., Szymonski M. (2017). AFM-Based Detection of Glycocalyx Degradation and Endothelial Stiffening in the Db/Db Mouse Model of Diabetes. Sci. Rep..

[8] Alvarez I.A., Lee M., Eshaq R.S., Leskova W., Harris N.R. (2024). High Glucose Induces Oxidative Stress That Alters Glycocalyx Proteoglycan Levels in Primary Rat Retinal Microvascular Endothelial Cells and in Isolated Ophthalmic Arteries. Pathophysiology.

[9] Van Den Berg B.M., Spaan J.A.E., Rolf T.M., Vink H. (2006). Atherogenic Region and Diet Diminish Glycocalyx Dimension and Increase Intima-to-Media Ratios at Murine Carotid Artery Bifurcation. Am. J. Physiol.-Heart Circ. Physiol..

[10] Eskens B.J.M., Leurgans T.M., Vink H., VanTeeffelen J.W.G.E. (2014). Early Impairment of Skeletal Muscle Endothelial Glycocalyx Barrier Properties in Diet-Induced Obesity in Mice. Physiol. Rep..

[11] Van Haare J., Kooi M.E., Vink H., Post M.J., Van Teeffelen J.W.G.E., Slenter J., Munts C., Cobelens H., Strijkers G.J., Koehn D. (2015). Early Impairment of Coronary Microvascular Perfusion Capacity in Rats on a High Fat Diet. Cardiovasc. Diabetol..

[12] Franceković P., Gliemann L. (2023). Endothelial Glycocalyx Preservation—Impact of Nutrition and Lifestyle. Nutrients.

[13] Katsanaki E., Georgiopoulos G., Thymis J., Pavlidis G., Katogiannis K., Nikolaou P.E., Vlastos D., Parissis J., Lambadiari V., Ikonomidis I. (2026). Effects of Nutritional Supplements on Vascular Function: A Narrative Review. J. Clin. Med..

[14] Gao L., Lipowsky H.H. (2010). Composition of the Endothelial Glycocalyx and Its Relation to Its Thickness and Diffusion of Small Solutes. Microvasc. Res..

[15] Florian J.A., Kosky J.R., Ainslie K., Pang Z., Dull R.O., Tarbell J.M. (2003). Heparan Sulfate Proteoglycan Is a Mechanosensor on Endothelial Cells. Circ. Res..

[16] Pahakis M.Y., Kosky J.R., Dull R.O., Tarbell J.M. (2007). The Role of Endothelial Glycocalyx Components in Mechanotransduction of Fluid Shear Stress. Biochem. Biophys. Res. Commun..

[17] Ebong E.E., Lopez-Quintero S.V., Rizzo V., Spray D.C., Tarbell J.M. (2014). Shear-Induced Endothelial NOS Activation and Remodeling via Heparan Sulfate, Glypican-1, and Syndecan-1. Integr. Biol..

[18] Voyvodic P.L., Min D., Liu R., Williams E., Chitalia V., Dunn A.K., Baker A.B. (2014). Loss of Syndecan-1 Induces a Pro-Inflammatory Phenotype in Endothelial Cells with a Dysregulated Response to Atheroprotective Flow. J. Biol. Chem..

[19] Yen W., Cai B., Yang J., Zhang L., Zeng M., Tarbell J.M., Fu B.M. (2015). Endothelial Surface Glycocalyx Can Regulate Flow-Induced Nitric Oxide Production in Microvessels In Vivo. PLoS ONE.

[20] Kumagai R., Lu X., Kassab G.S. (2009). Role of Glycocalyx in Flow-Induced Production of Nitric Oxide and Reactive Oxygen Species. Free Radic. Biol. Med..

[21] Psefteli P.-M., Kitscha P., Vizcay G., Fleck R., Chapple S.J., Mann G.E., Fowler M., Siow R.C. (2021). Glycocalyx Sialic Acids Regulate Nrf2-Mediated Signaling by Fluid Shear Stress in Human Endothelial Cells. Redox Biol..

[22] Singh A., Ramnath R.D., Foster R.R., Wylie E.C., Fridén V., Dasgupta I., Haraldsson B., Welsh G.I., Mathieson P.W., Satchell S.C. (2013). Reactive Oxygen Species Modulate the Barrier Function of the Human Glomerular Endothelial Glycocalyx. PLoS ONE.

[23] Schmidt E.P., Yang Y., Janssen W.J., Gandjeva A., Perez M.J., Barthel L., Zemans R.L., Bowman J.C., Koyanagi D.E., Yunt Z.X. (2012). The Pulmonary Endothelial Glycocalyx Regulates Neutrophil Adhesion and Lung Injury during Experimental Sepsis. Nat. Med..

[24] Ko K., Suzuki T., Ishikawa R., Hattori N., Ito R., Umehara K., Furihata T., Dohmae N., Linhardt R.J., Igarashi K. (2020). Ischemic Stroke Disrupts the Endothelial Glycocalyx through Activation of proHPSE via Acrolein Exposure. J. Biol. Chem..

[25] Kennett E.C., Davies M.J. (2009). Glycosaminoglycans Are Fragmented by Hydroxyl, Carbonate, and Nitrogen Dioxide Radicals in a Site-Selective Manner: Implications for Peroxynitrite-Mediated Damage at Sites of Inflammation. Free Radic. Biol. Med..

[26] Santic R., Martinovic L., Kumric M., Pavlovic N., Martinovic D., Jukic L., Pogorelic Z., Bozic J. (2026). Endothelial Mitochondrial Dysfunction in INOCA and Coronary Microvascular Dysfunction: Mechanisms, Sex Differences, and Therapeutic Implications. J. Cardiovasc. Dev. Dis..

[27] Mulivor A.W., Lipowsky H.H. (2009). Inhibition of Glycan Shedding and Leukocyte-Endothelial Adhesion in Postcapillary Venules by Suppression of Matrixmetalloprotease Activity with Doxycycline. Microcirculation.

[28] Augenreich M.A., Ferreira-Santos L., Power G., Ramirez-Perez F.I., Gonzalez-Vallejo J.D., Williams M.B., Imkaew N., Cho M.J., Wheeler A.A., Manrique-Acevedo C. (2026). ADAM17-Induced Shedding of Glypican-1 as a Mechanism of Impaired Endothelial Shear Stress Mechanotransduction. Am. J. Physiol.-Cell Physiol..

[29] Power G., Cho M.J., Beltran-Ornelas J.H., Augenreich M.A., Imkaew N., Lateef O.M., Ramirez-Perez F.I., Ferreira-Santos L., Manrique-Acevedo C., Martinez-Lemus L.A. (2026). Endothelial CD44 Cleavage by ADAM17 Impairs Shear Stress Mechanotransduction. Function.

[30] Zeng Y., Liu X.-H., Tarbell J., Fu B. (2015). Sphingosine 1-Phosphate Induced Synthesis of Glycocalyx on Endothelial Cells. Exp. Cell Res..

[31] Zeng Y., Adamson R.H., Curry F.-R.E., Tarbell J.M. (2014). Sphingosine-1-Phosphate Protects Endothelial Glycocalyx by Inhibiting Syndecan-1 Shedding. Am. J. Physiol.-Heart Circ. Physiol..

[32] Yang Y., Haeger S.M., Suflita M.A., Zhang F., Dailey K.L., Colbert J.F., Ford J.A., Picon M.A., Stearman R.S., Lin L. (2017). Fibroblast Growth Factor Signaling Mediates Pulmonary Endothelial Glycocalyx Reconstitution. Am. J. Respir. Cell Mol. Biol..

[33] Drost C.C., Rovas A., Kusche-Vihrog K., Van Slyke P., Kim H., Hoang V.C., Maynes J.T., Wennmann D.O., Pavenstädt H., Linke W. (2019). Tie2 Activation Promotes Protection and Reconstitution of the Endothelial Glycocalyx in Human Sepsis. Thromb. Haemost..

[34] Gouverneur M., Spaan J.A.E., Pannekoek H., Fontijn R.D., Vink H. (2006). Fluid Shear Stress Stimulates Incorporation of Hyaluronan into Endothelial Cell Glycocalyx. Am. J. Physiol.-Heart Circ. Physiol..

[35] Wang G., Kostidis S., Tiemeier G.L., Sol W.M.P.J., De Vries M.R., Giera M., Carmeliet P., Van Den Berg B.M., Rabelink T.J. (2020). Shear Stress Regulation of Endothelial Glycocalyx Structure Is Determined by Glucobiosynthesis. Arterioscler. Thromb. Vasc. Biol..

[36] Potter D.R., Damiano E.R. (2008). The Hydrodynamically Relevant Endothelial Cell Glycocalyx Observed In Vivo Is Absent In Vitro. Circ. Res..

[37] Jacob M., Rehm M., Loetsch M., Paul J.O., Bruegger D., Welsch U., Conzen P., Becker B.F. (2007). The Endothelial Glycocalyx Prefers Albumin for Evoking Shear Stress-Induced, Nitric Oxide-Mediated Coronary Dilatation. J. Vasc. Res..

[38] Sims D.E., Horne M.M. (1993). Non-Aqueous Fixative Preserves Macromolecules on the Endothelial Cell Surface: An in Situ Study. Eur. J. Morphol..

[39] Betteridge K.B., Arkill K.P., Neal C.R., Harper S.J., Foster R.R., Satchell S.C., Bates D.O., Salmon A.H.J. (2017). Sialic Acids Regulate Microvessel Permeability, Revealed by Novel in Vivo Studies of Endothelial Glycocalyx Structure and Function. J. Physiol..

[40] Giergiel M., Malek-Zietek K.E., Konior J., Targosz-Korecka M. (2021). Endothelial Glycocalyx Detection and Characterization by Means of Atomic Force Spectroscopy: Comparison of Various Data Analysis Approaches. Micron.

[41] Nieuwdorp M., Meuwese M.C., Mooij H.L., Ince C., Broekhuizen L.N., Kastelein J.J.P., Stroes E.S.G., Vink H. (2008). Measuring Endothelial Glycocalyx Dimensions in Humans: A Potential Novel Tool to Monitor Vascular Vulnerability. J. Appl. Physiol..

[42] Rovas A., Sackarnd J., Rossaint J., Kampmeier S., Pavenstädt H., Vink H., Kümpers P. (2021). Identification of Novel Sublingual Parameters to Analyze and Diagnose Microvascular Dysfunction in Sepsis: The NOSTRADAMUS Study. Crit. Care.

[43] Valerio L., Peters R.J., Zwinderman A.H., Pinto-Sietsma S.-J. (2019). Reproducibility of Sublingual Microcirculation Parameters Obtained from Sidestream Darkfield Imaging. PLoS ONE.

[44] Eickhoff M.K., Winther S.A., Hansen T.W., Diaz L.J., Persson F., Rossing P., Frimodt-Møller M. (2020). Assessment of the Sublingual Microcirculation with the GlycoCheck System: Reproducibility and Examination Conditions. PLoS ONE.

[45] Haldenby K.A., Chappell D.C., Winlove P., Parker K.H., Firth A. (2008). Focal and Regional Variations in the Composition of the Glycocalyx of Large Vessel Endothelium. J. Vasc. Res..

[46] Adepu S., Rosman C.W.K., Dam W., Van Dijk M.C.R.F., Navis G., Van Goor H., Bakker S.J.L., Van Den Born J. (2015). Incipient Renal Transplant Dysfunction Associates with Tubular Syndecan-1 Expression and Shedding. Am. J. Physiol.-Ren. Physiol..

[47] Hahn R.G., Zdolsek M., Zdolsek J. (2021). Plasma Concentrations of Syndecan-1 Are Dependent on Kidney Function. Acta Anaesthesiol. Scand..

[48] Weigel J.A., Raymond R.C., McGary C., Singh A., Weigel P.H. (2003). A Blocking Antibody to the Hyaluronan Receptor for Endocytosis (HARE) Inhibits Hyaluronan Clearance by Perfused Liver. J. Biol. Chem..

[49] Yanase F., Naorungroj T., Bellomo R. (2020). Glycocalyx Damage Biomarkers in Healthy Controls, Abdominal Surgery, and Sepsis: A Scoping Review. Biomarkers.

[50] Nicolescu A., Babotă M., Barros L., Rocchetti G., Lucini L., Tanase C., Mocan A., Bunea C.I., Crișan G. (2023). Bioaccessibility and Bioactive Potential of Different Phytochemical Classes from Nutraceuticals and Functional Foods. Front. Nutr..

[51] Hollman P.C.H., Bijsman M.N.C.P., Van Gameren Y., Cnossen E.P.J., De Vries J.H.M., Katan M.B. (1999). The Sugar Moiety Is a Major Determinant of the Absorption of Dietary Flavonoid Glycosides in Man. Free Radic. Res..

[52] Graefe E.U., Wittig J., Mueller S., Riethling A., Uehleke B., Drewelow B., Pforte H., Jacobasch G., Derendorf H., Veit M. (2001). Pharmacokinetics and Bioavailability of Quercetin Glycosides in Humans. J. Clin. Pharma.

[53] Vissers M.N., Zock P.L., Roodenburg A.J.C., Leenen R., Katan M.B. (2002). Olive Oil Phenols Are Absorbed in Humans. J. Nutr..

[54] Miró-Casas E., Covas M.-I., Fitó M., Farré-Albadalejo M., Marrugat J., De La Torre R. (2003). Tyrosol and Hydroxytyrosol Are Absorbed from Moderate and Sustained Doses of Virgin Olive Oil in Humans. Eur. J. Clin. Nutr..

[55] Mullen W., Edwards C.A., Crozier A. (2006). Absorption, Excretion and Metabolite Profiling of Methyl-, Glucuronyl-, Glucosyl- and Sulpho-Conjugates of Quercetin in Human Plasma and Urine after Ingestion of Onions. Br. J. Nutr..

[56] Stalmach A., Troufflard S., Serafini M., Crozier A. (2009). Absorption, Metabolism and Excretion of Choladi Green Tea Flavan-3-ols by Humans. Mol. Nutr. Food Res..

[57] Ottaviani J.I., Borges G., Momma T.Y., Spencer J.P.E., Keen C.L., Crozier A., Schroeter H. (2016). The Metabolome of [2-14C](−)-Epicatechin in Humans: Implications for the Assessment of Efficacy, Safety and Mechanisms of Action of Polyphenolic Bioactives. Sci. Rep..

[58] Czank C., Cassidy A., Zhang Q., Morrison D.J., Preston T., Kroon P.A., Botting N.P., Kay C.D. (2013). Human Metabolism and Elimination of the Anthocyanin, Cyanidin-3-Glucoside: A 13C-Tracer Study. Am. J. Clin. Nutr..

[59] De Ferrars R.M., Czank C., Zhang Q., Botting N.P., Kroon P.A., Cassidy A., Kay C.D. (2014). The Pharmacokinetics of Anthocyanins and Their Metabolites in Humans. Br. J. Pharmacol..

[60] Tomás-Barberán F.A., García-Villalba R., González-Sarrías A., Selma M.V., Espín J.C. (2014). Ellagic Acid Metabolism by Human Gut Microbiota: Consistent Observation of Three Urolithin Phenotypes in Intervention Trials, Independent of Food Source, Age, and Health Status. J. Agric. Food Chem..

[61] Šantić R., Kumrić M., Vilović M., Pavlović N., Rušić D., Bukić J., Božić J. (2026). How Gut Microbes Transform Dietary Phytonutrients: Enzymatic Pathways and Human Metabolite Exposure. Molecules.

[62] Walle T., Hsieh F., DeLegge M.H., Oatis J.E., Walle U.K. (2004). High Absorption but Very Low Bioavailability of Oral Resveratrol in Humans. Drug Metab. Dispos..

[63] Boocock D.J., Faust G.E.S., Patel K.R., Schinas A.M., Brown V.A., Ducharme M.P., Booth T.D., Crowell J.A., Perloff M., Gescher A.J. (2007). Phase I Dose Escalation Pharmacokinetic Study in Healthy Volunteers of Resveratrol, a Potential Cancer Chemopreventive Agent. Cancer Epidemiol. Biomark. Prev..

[64] Cova D., De Angelis L., Giavarini F., Palladini G., Perego R. (1992). Pharmacokinetics and Metabolism of Oral Diosmin in Healthy Volunteers. Int. J. Clin. Pharmacol. Ther. Toxicol..

[65] Garner R.C., Garner J.V., Gregory S., Whattam M., Calam A., Leong D. (2002). Comparison of the Absorption of Micronized (Daflon 500^®^ Mg) and Nonmicronized 14C-diosmin Tablets After Oral Administration to Healthy Volunteers by Accelerator Mass Spectrometry and Liquid Scintillation Counting. J. Pharm. Sci..

[66] Russo R., Chandradhara D., De Tommasi N. (2018). Comparative Bioavailability of Two Diosmin Formulations after Oral Administration to Healthy Volunteers. Molecules.

[67] Vareed S.K., Kakarala M., Ruffin M.T., Crowell J.A., Normolle D.P., Djuric Z., Brenner D.E. (2008). Pharmacokinetics of Curcumin Conjugate Metabolites in Healthy Human Subjects. Cancer Epidemiol. Biomark. Prev..

[68] Flory S., Sus N., Haas K., Jehle S., Kienhöfer E., Waehler R., Adler G., Venturelli S., Frank J. (2021). Increasing Post-Digestive Solubility of Curcumin Is the Most Successful Strategy to Improve Its Oral Bioavailability: A Randomized Cross-Over Trial in Healthy Adults and In Vitro Bioaccessibility Experiments. Mol. Nutr. Food Res..

[69] Fança-Berthon P., Tenon M., Bouter-Banon S.L., Manfré A., Maudet C., Dion A., Chevallier H., Laval J., Van Breemen R.B. (2021). Pharmacokinetics of a Single Dose of Turmeric Curcuminoids Depends on Formulation: Results of a Human Crossover Study. J. Nutr..

[70] Grafeneder J., Derhaschnig U., Eskandary F., Buchtele N., Sus N., Frank J., Jilma B., Schoergenhofer C. (2022). Micellar Curcumin: Pharmacokinetics and Effects on Inflammation Markers and PCSK-9 Concentrations in Healthy Subjects in a Double-Blind, Randomized, Active-Controlled, Crossover Trial. Mol. Nutr. Food Res..

[71] Vermeulen M., Klöpping-Ketelaars I.W.A.A., Van Den Berg R., Vaes W.H.J. (2008). Bioavailability and Kinetics of Sulforaphane in Humans after Consumption of Cooked versus Raw Broccoli. J. Agric. Food Chem..

[72] Egner P.A., Chen J.G., Wang J.B., Wu Y., Sun Y., Lu J.H., Zhu J., Zhang Y.H., Chen Y.S., Friesen M.D. (2011). Bioavailability of Sulforaphane from Two Broccoli Sprout Beverages: Results of a Short-Term, Cross-over Clinical Trial in Qidong, China. Cancer Prev. Res..

[73] Kopplin G., Rokstad A.M., Mélida H., Bulone V., Skjåk-Bræk G., Aachmann F.L. (2018). Structural Characterization of Fucoidan from *Laminaria hyperborea*: Assessment of Coagulation and Inflammatory Properties and Their Structure–Function Relationship. ACS Appl. Bio Mater..

[74] Ke S., Wei B., Qiu W., Zhou T., Wang S., Chen J., Chen J., Zhang H., Jin W., Wang H. (2020). Structural Characterization and *α* -Glucosidase Inhibitory and Antioxidant Activities of Fucoidans Extracted from *Saccharina Japonica*. Chem. Biodivers..

[75] Zayed A., El-Aasr M., Ibrahim A.-R.S., Ulber R. (2020). Fucoidan Characterization: Determination of Purity and Physicochemical and Chemical Properties. Mar. Drugs.

[76] Irhimeh M.R., Fitton J.H., Lowenthal R.M., Kongtawelert P. (2005). A Quantitative Method to Detect Fucoidan in Human Plasma Using Anovel Antibody. Methods Find. Exp. Clin. Pharmacol..

[77] Kadena K., Tomori M., Iha M., Nagamine T. (2018). Absorption Study of Mozuku Fucoidan in Japanese Volunteers. Mar. Drugs.

[78] Huang D., Ou B., Prior R.L. (2005). The Chemistry behind Antioxidant Capacity Assays. J. Agric. Food Chem..

[79] Long L.H., Clement M.V., Halliwell B. (2000). Artifacts in Cell Culture: Rapid Generation of Hydrogen Peroxide on Addition of (−)-Epigallocatechin, (−)-Epigallocatechin Gallate, (+)-Catechin, and Quercetin to Commonly Used Cell Culture Media. Biochem. Biophys. Res. Commun..

[80] Dinkova-Kostova A.T., Holtzclaw W.D., Cole R.N., Itoh K., Wakabayashi N., Katoh Y., Yamamoto M., Talalay P. (2002). Direct Evidence That Sulfhydryl Groups of Keap1 Are the Sensors Regulating Induction of Phase 2 Enzymes That Protect against Carcinogens and Oxidants. Proc. Natl. Acad. Sci. USA.

[81] Nelson K.M., Dahlin J.L., Bisson J., Graham J., Pauli G.F., Walters M.A. (2017). The Essential MedicinalChemistry of Curcumin. J. Med. Chem..

[82] Pugh N.D., Tamta H., Balachandran P., Wu X., Howell J., Dayan F.E., Pasco D.S. (2008). The Majority of in Vitro Macrophage Activation Exhibited by Extracts of Some Immune Enhancing Botanicals Is Due to Bacterial Lipoproteins and Lipopolysaccharides. Int. Immunopharmacol..

[83] Wang J., Zhang Q., Zhang Z., Zhang H., Niu X. (2010). Structural Studies on a Novel Fucogalactan Sulfate Extracted from the Brown Seaweed Laminaria Japonica. Int. J. Biol. Macromol..

[84] Lee J.-B., Koizumi S., Hayashi K., Hayashi T. (2010). Structure of Rhamnan Sulfate from the Green Alga Monostroma Nitidum and Its Anti-Herpetic Effect. Carbohydr. Polym..

[85] Marinval N., Saboural P., Haddad O., Maire M., Bassand K., Geinguenaud F., Djaker N., Ben Akrout K., Lamy De La Chapelle M., Robert R. (2016). Identification of a Pro-Angiogenic Potential and Cellular Uptake Mechanism of a LMW Highly Sulfated Fraction of Fucoidan from Ascophyllum Nodosum. Mar. Drugs.

[86] Leite E.L., Medeiros M.G.L., Rocha H.A.O., Farias G.G.M., Da Silva L.F., Chavante S.F., De Abreu L.D., Dietrich C.P., Nader H.B. (1998). Structure and Pharmacological Activities of a Sulfated Xylofucoglucuronan from the Alga Spatoglossum Schröederi. Plant Sci..

[87] De Oliveira Rocha H.A., De Medeiros Bezerra L.C., De Albuquerque I.R., Costa L.S., Guerra C.M., De Abreu L.D., Nader H.B., Leite E.L. (2005). A Xylogalactofucan from the Brown Seaweed *Spatoglossum schröederi* Stimulates the Synthesis of an Antithrombotic Heparan Sulfate from Endothelial Cells. Planta Med..

[88] Rocha H.A.O., Moraes F.A., Trindade E.S., Franco C.R.C., Torquato R.J.S., Veiga S.S., Valente A.P., Mourão P.A.S., Leite E.L., Nader H.B. (2005). Structural and Hemostatic Activities of a Sulfated Galactofucan from the Brown Alga Spatoglossum Schroederi. J. Biol. Chem..

[89] Barroso E., Costa L., Medeiros V., Cordeiro S., Costa M., Franco C., Nader H., Leite E., Rocha H. (2008). A Non-Anticoagulant Heterofucan Has Antithrombotic Activity in Vivo. Planta Med..

[90] Soeda S., Fujii N., Shimeno H. (1995). Atsuo Nagamatsu Oversulfated Fucoidan and Heparin Suppress Endotoxin Induction of Plasminogen Activator Inhibitor-1 in Cultured Human Endothelial Cells: Their Possible Mechanism of Action. Biochim. Biophys. Acta (BBA)-Mol. Cell Res..

[91] Soeda S., Kozako T., Iwata K., Shimeno H. (2000). Oversulfated Fucoidan Inhibits the Basic Fibroblast Growth Factor-Induced Tube Formation by Human Umbilical Vein Endothelial Cells: Its Possible Mechanism of Action. Biochim. Biophys. Acta (BBA)-Mol. Cell Res..

[92] Chabut D., Fischer A.-M., Colliec-Jouault S., Laurendeau I., Matou S., Le Bonniec B., Helley D. (2003). Low Molecular Weight Fucoidan and Heparin Enhance the Basic Fibroblast Growth Factor-Induced Tube Formation of Endothelial Cells through Heparan Sulfate-Dependent A6 Overexpression. Mol. Pharmacol..

[93] Lake A.C., Vassy R., Di Benedetto M., Lavigne D., Le Visage C., Perret G.Y., Letourneur D. (2006). Low Molecular Weight Fucoidan Increases VEGF165-Induced Endothelial Cell Migration by Enhancing VEGF165 Binding to VEGFR-2 and NRP1. J. Biol. Chem..

[94] Haddad O., Guyot E., Marinval N., Chevalier F., Maillard L., Gadi L., Laguillier-Morizot C., Oudar O., Sutton A., Charnaux N. (2015). Heparanase and Syndecan-4 Are Involved in Low Molecular Weight Fucoidan-Induced Angiogenesis. Mar. Drugs.

[95] Yuan L., Cheng S., Sol W.M.P.J., Van Der Velden A.I.M., Vink H., Rabelink T.J., Van Den Berg B.M. (2022). Heparan Sulfate Mimetic Fucoidan Restores the Endothelial Glycocalyx and Protects against Dysfunction Induced by Serum of COVID-19 Patients in the Intensive Care Unit. ERJ Open Res..

[96] Regier M., Drost C.C., Rauen M., Pavenstädt H., Rovas A., Kümpers P., Vink H., Long R.M., Linke W.A., Nofer J.-R. (2022). A Dietary Supplement Containing Fucoidan Preserves Endothelial Glycocalyx through ERK/MAPK Signaling and Protects against Damage Induced by CKD Serum. Int. J. Mol. Sci..

[97] Li Z., Wu N., Wang J., Yue Y., Geng L., Zhang Q. (2024). Low Molecular Weight Fucoidan Restores Diabetic Endothelial Glycocalyx by Targeting Neuraminidase2: A New Therapy Target in Glycocalyx Shedding. Br. J. Pharmacol..

[98] Buijsers B., Maciej-Hulme M., Jacobs M., Bebber M.B., De Graaf M., Salmenov R., Parr N., Rabelink T.J., Nijenhuis T., Van Der Vlag J. (2023). Glycosaminoglycans and Fucoidan Have a Protective Effect on Experimental Glomerulonephritis. Front. Mol. Biosci..

[99] Fernandes P.L.M., De Castro Silva P.A., Miranda C.H. (2026). Fucoidan Preserves Endothelial Glycocalyx and Reduces Microvascular Obstruction after Myocardial Ischemia-Reperfusion. Microvasc. Res..

[100] Okamoto T., Akita N., Terasawa M., Hayashi T., Suzuki K. (2019). Rhamnan Sulfate Extracted from Monostroma Nitidum Attenuates Blood Coagulation and Inflammation of Vascular Endothelial Cells. J. Nat. Med..

[101] Terasawa M., Hiramoto K., Uchida R., Suzuki K. (2022). Anti-Inflammatory Activity of Orally Administered Monostroma Nitidum Rhamnan Sulfate against Lipopolysaccharide-Induced Damage to Mouse Organs and Vascular Endothelium. Mar. Drugs.

[102] Patil N.P., Gómez-Hernández A., Zhang F., Cancel L., Feng X., Yan L., Xia K., Takematsu E., Yang E.Y., Le V. (2022). Rhamnan Sulfate Reduces Atherosclerotic Plaque Formation and Vascular Inflammation. Biomaterials.

[103] Terasawa M., Zang L., Hiramoto K., Shimada Y., Mitsunaka M., Uchida R., Nishiura K., Matsuda K., Nishimura N., Suzuki K. (2023). Oral Administration of Rhamnan Sulfate from Monostroma Nitidum Suppresses Atherosclerosis in ApoE-Deficient Mice Fed a High-Fat Diet. Cells.

[104] Yamamoto C., Shimada S., Fujiwara Y., Lee J.-B., Hayashi T., Kaji T. (2005). Proteoglycans Released from Cultured Bovine Aortic Endothelial Cell Layers by Sodium Spirulan Are Both Perlecan and Biglycan. Biol. Pharm. Bull..

[105] Nagamine T., Nakazato K., Tomioka S., Iha M., Nakajima K. (2014). Intestinal Absorption of Fucoidan Extracted from the Brown Seaweed, Cladosiphon Okamuranus. Mar. Drugs.

[106] Tan J., Song Y., Wang J., Wu N., Yue Y., Zhang Q. (2023). Pharmacokinetics of Fucoidan and Low Molecular Weight Fucoidan from Saccharina Japonica after Oral Administration to Mice. J. Ocean. Limnol..

[107] Tokita Y., Hirayama M., Nakajima K., Tamaki K., Iha M., Nagamine T. (2017). Detection of Fucoidan in Urine after Oral Intake of Traditional Japanese Seaweed, *Okinawa Mozuku* (*Cladosiphon Okamuranus* Tokida). J. Nutr. Sci. Vitaminol..

[108] Šantić R., Martinović L., Pavlović N., Martinović D., Vrdoljak J., Kumrić M., Vilović M., Božić J. (2026). Natural Bioactive Compounds Targeting Gut Barrier Integrity and Metabolic Endotoxemia in Cardiometabolic Disease: Mechanistic Insights and Translational Perspectives. Molecules.

[109] Bao C.-J., Liu Y.-X., Li M.-X., Yi H., Li X.-T., Chang X.-W., Zhang W.-J., Qin X.-J., Geng Y.-Q., Duan J.-L. (2026). Functional Reinterpretation of Food-Medicine Homologous Polysaccharides: From Bioactive Macromolecules to Multifunctional Materials. Food Med. Homol..

[110] Liu Y., Tao X., Wang X., Sun Z., Shen P., Li W., Li X., Li P. (2026). Fucoidan Improves Glucose and Lipid Metabolism in T2DM Rats through the Gut-Liver Axis. Food Sci. Hum. Wellness.

[111] Peters W., Kusche-Vihrog K., Oberleithner H., Schillers H. (2015). Cystic Fibrosis Transmembrane Conductance Regulator Is Involved in Polyphenol-Induced Swelling of the Endothelial Glycocalyx. Nanomed. Nanotechnol. Biol. Med..

[112] Cutler B.R., Gholami S., Chua J.S., Kuberan B., Anandh Babu P.V. (2018). Blueberry Metabolites Restore Cell Surface Glycosaminoglycans and Attenuate Endothelial Inflammation in Diabetic Human Aortic Endothelial Cells. Int. J. Cardiol..

[113] Kalea A.Z., Lamari F.N., Theocharis A.D., Cordopatis P., Schuschke D.A., Karamanos N.K., Klimis-Zacas D.J. (2006). Wild Blueberry (Vaccinium Angustifolium) Consumption Affects the Composition and Structure of Glycosaminoglycans in Sprague-Dawley Rat Aorta. J. Nutr. Biochem..

[114] Kristo A.S., Malavaki C.J., Lamari F.N., Karamanos N.K., Klimis-Zacas D. (2012). Wild Blueberry (V. Angustifolium)-Enriched Diets Alter Aortic Glycosaminoglycan Profile in the Spontaneously Hypertensive Rat. J. Nutr. Biochem..

[115] Peters W., Drüppel V., Kusche-Vihrog K., Schubert C., Oberleithner H. (2012). Nanomechanics and Sodium Permeability of Endothelial Surface Layer Modulated by Hawthorn Extract WS 1442. PLoS ONE.

[116] Mitra R., Nersesyan A., Pentland K., Melin M.M., Levy R.M., Ebong E.E. (2022). Diosmin and Its Glycocalyx Restorative and anti-inflammatory Effects on Injured Blood Vessels. FASEB J..

[117] Żurawek D., Pydyn N., Major P., Szade K., Trzos K., Kuś E., Pośpiech E., Małczak P., Radkowiak D., Budzyński A. (2025). Diosmetin Alleviates TNFα-Induced Liver Inflammation by Improving Liver Sinusoidal Endothelial Cell Dysfunction. Biomed. Pharmacother..

[118] Wu H., Chen Z., Chen J.-Z., Xie J., Xu B. (2018). Resveratrol Improves Tube Formation in AGE-Induced Late Endothelial Progenitor Cells by Suppressing Syndecan-4 Shedding. Oxidative Med. Cell. Longev..

[119] Abu El-Asrar A.M., Nawaz M.I., Ahmad A., Siddiquei M., Allegaert E., Gikandi P.W., De Hertogh G., Opdenakker G. (2026). Dysregulated Hyaluronan Metabolism Drives Inflammation and Angiogenesis in Proliferative Diabetic Retinopathy. Front. Immunol..

[120] Nugent W.H., Carr D.A., Friedman J., Song B.K. (2023). Novel Transdermal Curcumin Therapeutic Preserves Endothelial Barrier Function in a High-Dose LPS Rat Model. Artif. Cells Nanomed. Biotechnol..

[121] Viudes D.R., Mateus A.R., Silva C.A., Do Carmo Franco M. (2026). Curcuma Longa Improves Endothelial Glycocalyx Integrity and Redox-Inflammatory Pathways in Type 2 Diabetes Mellitus: A Randomized Double-Blind Placebo-Controlled Study. Eur. J. Nutr..

[122] Zhang D., Han S., Zhou Y., Qi B., Wang X. (2020). Therapeutic Effects of Mangiferin on Sepsis-Associated Acute Lung and Kidney Injuries via the Downregulation of Vascular Permeability and Protection of Inflammatory and Oxidative Damages. Eur. J. Pharm. Sci..

[123] Zhang D., Qi B., Zhu W.-W., Huang X., Wang X. (2020). Crocin Alleviates Lipopolysaccharide-Induced Acute Respiratory Distress Syndrome by Protecting against Glycocalyx Damage and Suppressing Inflammatory Signaling Pathways. Inflamm. Res..

[124] Kong G., Huang X., Wang L., Li Y., Sun T., Han S., Zhu W., Ma M., Xu H., Li J. (2016). Astilbin Alleviates LPS-Induced ARDS by Suppressing MAPK Signaling Pathway and Protecting Pulmonary Endothelial Glycocalyx. Int. Immunopharmacol..

[125] Liu X.-Y., Xu H.-X., Li J.-K., Zhang D., Ma X.-H., Huang L.-N., Lü J.-H., Wang X.-Z. (2018). Neferine Protects Endothelial Glycocalyx via Mitochondrial ROS in Lipopolysaccharide-Induced Acute Respiratory Distress Syndrome. Front. Physiol..

[126] Huang L., Zhang X., Ma X., Zhang D., Li D., Feng J., Pan X., Lü J., Wang X., Liu X. (2018). Berberine Alleviates Endothelial Glycocalyx Degradation and Promotes Glycocalyx Restoration in LPS-Induced ARDS. Int. Immunopharmacol..

[127] Yang H., Zhu L., Gu Y., Kong X., Liu Y., Chen M., Xie X., Luo J., Chen S. (2019). Berberine Inhibits Low Shear Stress-Induced Glycocalyx Degradation via Modulating AMPK and P47phox/Hyal2 Signal Pathway. Eur. J. Pharmacol..

[128] Zhu T., Wang H., Wang L., Zhong X., Huang W., Deng X., Guo H., Xiong J., Xu Y., Fan J. (2019). Ginsenoside Rg1 Attenuates High Glucose-induced Endothelial Barrier Dysfunction in Human Umbilical Vein Endothelial Cells by Protecting the Endothelial Glycocalyx. Exp. Ther. Med..

[129] Qiao Y., Yokoyama M., Kameyama K., Asano G. (1993). Effect of Vitamin E on Vascular Integrity in Cholesterol-Fed Guinea Pigs. Arterioscler. Thromb..

[130] Fisher E.J., McLennan S.V., Yue D.K., Turtle J.R. (1994). Cell-Associated Proteoglycans of Retinal Pericytes and Endothelial Cells: Modulation by Glucose and Ascorbic Acid. Microvasc. Res..

[131] Madokoro Y., Kamikokuryo C., Niiyama S., Ito T., Hara S., Ichinose H., Kakihana Y. (2022). Early Ascorbic Acid Administration Prevents Vascular Endothelial Cell Damage in Septic Mice. Front. Pharmacol..

[132] Belousoviene E., Pranskuniene Z., Vaitkaitiene E., Pilvinis V., Pranskunas A. (2023). Effect of High-Dose Intravenous Ascorbic Acid on Microcirculation and Endothelial Glycocalyx during Sepsis and Septic Shock: A Double-Blind, Randomized, Placebo-Controlled Study. BMC Anesthesiol..

[133] Qiao X., Kashiouris M.G., L’Heureux M., Fisher B.J., Leichtle S.W., Truwit J.D., Nanchal R., Hite R.D., Morris P.E., Martin G.S. (2022). Biological Effects of Intravenous Vitamin C on Neutrophil Extracellular Traps and the Endothelial Glycocalyx in Patients with Sepsis-Induced ARDS. Nutrients.

[134] Duan W., Paka L., Pillarisetti S. (2005). Distinct Effects of Glucose and Glucosamine on Vascular Endothelial and Smooth Muscle Cells: Evidence for a Protective Role for Glucosamine in Atherosclerosis. Cardiovasc. Diabetol..

[135] Persiani S., Roda E., Rovati L.C., Locatelli M., Giacovelli G., Roda A. (2005). Glucosamine Oral Bioavailability and Plasma Pharmacokinetics after Increasing Doses of Crystalline Glucosamine Sulfate in Man. Osteoarthr. Cartil..

[136] Kalea A.Z., Lamari F.N., Theocharis A.D., Schuschke D.A., Karamanos N.K., Klimis-Zacas D.J. (2006). Dietary Manganese Affects the Concentration, Composition and Sulfation Pattern of Heparan Sulfate Glycosaminoglycans in Sprague-Dawley Rat Aorta. Biometals.

[137] Gemmel M., Sutton E.F., Brands J., Burnette L., Gallaher M.J., Powers R.W. (2021). l -Citrulline Supplementation during Pregnancy Improves Perinatal and Postpartum Maternal Vascular Function in a Mouse Model of Preeclampsia. Am. J. Physiol.-Regul. Integr. Comp. Physiol..

[138] Ichikawa S., Gohda T., Murakoshi M., Li Z., Adachi E., Koshida T., Suzuki Y. (2020). Aspartic Acid Supplementation Ameliorates Symptoms of Diabetic Kidney Disease in Mice. FEBS Open Bio.

[139] Justus A.C., Hoggatt A.M., Faulk W.P. (1995). Heparan-Dependent Endothelial Antithrombin Binding Is Increased by Butyrate. Thromb. Res..

[140] Ramasamy S., Boissonneault G.A., Lipke D.W., Hennig B. (1993). Proteoglycans and Endothelial Barrier Function: Effect of Linoleic Acid Exposure to Porcine Pulmonary Artery Endothelial Cells. Atherosclerosis.

[141] Gimblet C.J., Ernst J.W., Bell B., Bos K.D., Stroud A.K., Wendt L.H., Donato A.J., Jalal D.I., Pierce G.L. (2024). Effect of Glycocalyx-Targeted Therapy on Vascular Function in Older Adults: A Randomized Controlled Trial. J. Appl. Physiol..

[142] Gimblet C.J., Donato A.J., Jalal D.I., Pierce G.L. (2025). Sex Differences in Endothelial Glycocalyx Thickness and the Response to Glycocalyx-targeted Therapy among Older Adults. Physiol. Rep..

[143] Smith J.A., Ramirez-Perez F.I., Burr K., Gonzalez-Vallejo J.D., Morales-Quinones M., McMillan N.J., Ferreira-Santos L., Sharma N., Foote C.A., Martinez-Lemus L.A. (2024). Impact of Dietary Supplementation of Glycocalyx Precursors on Vascular Function in Type 2 Diabetes. J. Appl. Physiol..

[144] Van Der Velden A.I.M., IJpelaar D.H.T., Chandie Shaw P.K., Pijl H., Vink H., Van Der Vlag J., Rabelink T.J., Van Den Berg B.M. (2024). Role of Dietary Interventions on Microvascular Health in South-Asian Surinamese People with Type 2 Diabetes in the Netherlands: A Randomized Controlled Trial. Nutr. Diabetes.

[145] Ikonomidis I., Katsanaki E., Thymis J., Pavlidis G., Lampadaki K., Katogiannis K., Vaiopoulos A., Lazarou V., Kostelli G., Michalopoulou E. (2024). The Effect of 4-Month Treatment with Glycocalyx Dietary Supplement on Endothelial Glycocalyx Integrity and Vascular Function in Patients with Psoriasis. Nutrients.

[146] Pavlidis G., Kountouri A., Katogiannis K., Thymis J., Nikolaou P.E., Chania C., Karalis J., Kostelli G., Michalopoulou E., Katsanaki E. (2025). Effects of 4-month Treatment with Glycocalyx Dietary Supplement on Endothelial Glycocalyx and Vascular Function after COVID-19 Infection. Eur. J. Clin. Investig..

[147] Biglari S., Yuan L., Mischak H., Siwy J., Latosinska A., Banasik M., Van Den Berg B.M. (2025). Dietary Glycocalyx Mimetic Reduces Vascular Risk in Type 2 Diabetes: Evidence from Urinary Peptidomic Classifiers in a South–Asian Surinamese Cohort. Diabetes Res. Clin. Pract..

[148] Ikonomidis I., Katogiannis K., Chania C., Iakovis N., Tsoumani M., Christodoulou A., Brinia E., Pavlidis G., Thymis J., Tsilivarakis D. (2023). Association of Hydroxytyrosol Enriched Olive Oil with Vascular Function in Chronic Coronary Disease. Eur. J. Clin. Investig..

[149] Vrentzos E., Ikonomidis I., Pavlidis G., Katogiannis K., Korakas E., Kountouri A., Pliouta L., Michalopoulou E., Pelekanou E., Boumpas D. (2024). Six-Month Supplementation with High Dose Coenzyme Q10 Improves Liver Steatosis, Endothelial, Vascular and Myocardial Function in Patients with Metabolic-Dysfunction Associated Steatotic Liver Disease: A Randomized Double-Blind, Placebo-Controlled Trial. Cardiovasc. Diabetol..

[150] Munguía L., Silva S., Villarreal F., Nájera N., Ceballos G. (2026). Effects of Cacao Flavonoids in Long COVID-19 Patients with Chronic Fatigue: FLALOC, a Placebo-Controlled Randomized Clinical Trial. J. Clin. Med..

[151] Pranskuniene Z., Belousoviene E., Baranauskiene N., Eimantas N., Vaitkaitiene E., Bernatoniene J., Brazaitis M., Pranskunas A. (2020). Modulation of Endothelial Glycocalyx and Microcirculation in Healthy Young Men during High-Intensity Sprint Interval Cycling-Exercise by Supplementation with Pomegranate Extract. A Randomized Controlled Trial. Int. J. Environ. Res. Public. Health.

[152] Stougaard E.B., Tougaard N.H., Sivalingam S., Hansen C.S., Størling J., Hansen T.W., Frimodt-Møller M., Steinert R.E., Varasteh S., Groop P.-H. (2024). Effects of Probiotics and Fibers on Markers of Nephropathy, Inflammation, Intestinal Barrier Dysfunction and Endothelial Dysfunction in Individuals with Type 1 Diabetes and Albuminuria. The ProFOS Study. J. Diabetes Its Complicat..

[153] Fancher I.S., Le Master E., Ahn S.J., Adamos C., Lee J.C., Berdyshev E., Dull R.O., Phillips S.A., Levitan I. (2020). Impairment of Flow-Sensitive Inwardly Rectifying K^+^ Channels via Disruption of Glycocalyx Mediates Obesity-Induced Endothelial Dysfunction. Arterioscler. Thromb. Vasc. Biol..

[154] Ahn S.J., Le Master E., Lee J.C., Phillips S.A., Levitan I., Fancher I.S. (2022). Differential Effects of Obesity on Visceral versus Subcutaneous Adipose Arteries: Role of Shear-Activated Kir2.1 and Alterations to the Glycocalyx. Am. J. Physiol.-Heart Circ. Physiol..

[155] Kang H., Sun A., Wu Q., Yang J., Zhang W., Qu Y., Gao M., Deng X. (2020). Atherogenic Diet-Diminished Endothelial Glycocalyx Contributes to Impaired Vasomotor Properties in Rat. Am. J. Physiol.-Heart Circ. Physiol..

[156] Zheng X., Deacon C., King A.J., Machin D.R. (2022). Microcirculatory and Glycocalyx Properties Are Lowered by High-Salt Diet but Augmented by Western Diet in Genetically Heterogeneous Mice. Am. J. Physiol.-Heart Circ. Physiol..

[157] Li Z., Sabouri M., Zheng X., Machin D.R. (2025). Time Course and Contributors to Greater Glycocalyx Thickness and Integrity Following Western Diet Consumption. Am. J. Physiol.-Heart Circ. Physiol..

[158] Sabouri M., Li Z., Zheng X., Machin D.R. (2025). Western Diet-Induced Endothelial Glycocalyx Remodeling Is Partially Reversed by Fasting. Am. J. Physiol.-Heart Circ. Physiol..

[159] Andersson M., Ågren A., Henriksson P., Wallén H., Thorell A. (2025). Influence of Bariatric Surgery on Endothelial and Glycocalyx Biomarkers in Obesity and Type 2 Diabetes. J. Clin. Endocrinol. Metab..

[160] Lamontagne F., Masse M.-H., Menard J., Sprague S., Pinto R., Heyland D.K., Cook D.J., Battista M.-C., Day A.G., Guyatt G.H. (2022). Intravenous Vitamin C in Adults with Sepsis in the Intensive Care Unit. N. Engl. J. Med..

[161] Halegoua-DeMarzio D., Navarro V., Ahmad J., Avula B., Barnhart H., Barritt A.S., Bonkovsky H.L., Fontana R.J., Ghabril M.S., Hoofnagle J.H. (2023). Liver Injury Associated with Turmeric—A Growing Problem: Ten Cases from the Drug-Induced Liver Injury Network [DILIN]. Am. J. Med..

